# Microbiome‐mediated plant‐soil feedbacks as a tool for resilient agriculture

**DOI:** 10.1002/imo2.70112

**Published:** 2026-07-03

**Authors:** Muhammad Asif, Hazrat Bilal, Min Xiao, Lei Dong, Wen‐Jun Li

**Affiliations:** ^1^ State Key Laboratory of Biocontrol, Guangdong Provincial Key Laboratory of Plant Stress Biology, School of Life Sciences Sun Yat‐sen University Guangzhou China

**Keywords:** ecosystem services, microbial inoculants, plant‐soil feedback, plant defense, root exudates, sustainable agriculture

## Abstract

Conventional agricultural intensification has eroded the biological foundations of productive soil by disrupting the reciprocal interactions, plant‐soil feedback (PSF), through which plants actively shape and are continuously shaped by the soil microbiome. Although the molecular mechanisms governing PSF direction are increasingly well‐characterized, deliberate PSF management as a practical tool for resilient crop production remains underdeveloped. This is largely because no integrative framework has translated these ecological insights into state‐contingent, field‐deployable decision rules. This review addresses that gap by first synthesizing the molecular basis of microbiome assembly, encompassing host genetic control of root exudate chemistry, receptor‐mediated microbial recognition, and the bidirectional chemical dialogue between plant and rhizosphere. Building from this mechanistic foundation, we move beyond cataloging mechanisms to present a novel, integrative three‐pillar management framework for actively steering PSF. This framework operationalizes PSF management by strategically combining: (i) engineering the plant partner (leveraging genetics to recruit beneficial microbiomes), (ii) managing the soil habitat (using agronomic practices to engineer suppressive ecological legacies), and (iii) deploying advanced microbial consortia. Critically, we argue that the recent meta‐omics revolution, integrating metagenomics, metatranscriptomics, metaproteomics, and metabolomics, is the enabling technology that transforms this framework from a conceptual model into a quantifiable, diagnosable, and agriculturally deployable system. Multi‐omics tools provide the empirical foundation for diagnosing soil feedback states, monitoring intervention outcomes, and building predictive models. By bridging the lab‐field gap and evaluating key translational challenges, this review provides a coherent, actionable roadmap for harnessing PSF to achieve cultivated resilience in modern agriculture.

## Highlights

- Plant‐soil feedback (PSF) governs microbial interactions vital for crop yield.
- Host genetic control of root exudates that govern microbiome assembly, enables the engineering of positive PSF.
- Steering positive PSF enhances nutrient cycling and suppresses soil pathogens.
- Synthesizes a pathway from lab to field by advocating for microbiome‐assisted breeding and diagnostic, adaptive management cycles to overcome the implementation gap.
- Meta‐omics leveraged, integrated state‐contingent agronomic and microbial interventions act as an achievable alternative to chemical input dependence.

## Graphical abstract

Translating plant‐soil feedbacks (PSFs) into reliable agricultural management requires shifting from input‐dependent practices toward endogenous, process‐driven crop resilience. To bridge this gap, we propose a predictive framework centered on three strategic interventions: engineering the crop holobiont, diversifying the soil habitat, and deploying targeted microbial consortia. Rather than relying on static correlations, this framework integrates field‐scale gradients with mechanistic models to validate soil legacies dynamically. By closing this empirical‐predictive loop, we can turn complex ecological principles into quantifiable, actionable designs for climate‐resilient cropping systems.

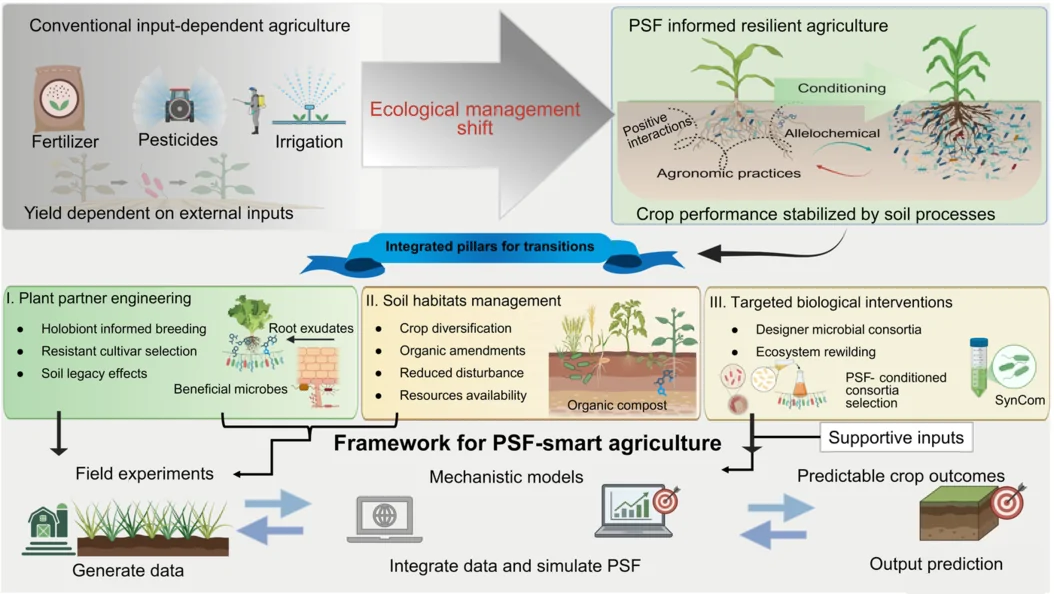

## 1 INTRODUCTION

Meeting the nutritional demands of a projected human population of 10 billion by 2060 requires a 50% increase in food production [1, 2]. Yet current intensive agricultural systems characterized by input‑dependent, low‑diversity monocultures [3] and often low‑yielding varieties are vulnerable and incur substantial ecological costs, including pathogen outbreaks [4], soil degradation [5], nutrient pollution [3], biodiversity loss [6, 7], and heightened susceptibility to climate‑induced stressors such as drought and extreme heat [8, 9]. There is a pressing need for agricultural frameworks that move beyond simplistic input‐output models to embrace the ecological complexity of agroecosystems, particularly the dynamic interactions between plants, soil, and microorganisms [10, 11].

The concept of plant‐soil feedbacks (PSF) provides a robust ecological framework for understanding these interactions. PSF encompasses the cyclical process whereby plants modify soil physicochemical and biological properties through root exudation, litter inputs, and microbiome recruitment, which in turn influence the performance of subsequent plants (Figure 1A−C) [12, 13, 14, 15]. These feedbacks operate across a spatial hierarchy, originating from molecular exchanges within the distinct zones of the rhizosphere, including the endo‐rhizosphere and rhizoplane, and scaling up to influence community‐level dynamics and farmland productivity (Figure 1A−C). This complexity demands a mechanistic understanding of PSF. For instance, plant litter decomposability modulates soil nutrient cycling, but the magnitude and direction of this feedback depend on the functional composition of decomposer and pathogen communities [16, 17, 18]. Such interplay underpins aboveground‐belowground linkages [19, 20]. In natural ecosystems, PSF fundamentally structures plant community composition, maintains diversity, and drives successional trajectories [14, 21, 22, 23]. Consequently, PSF can act as an evolutionary agent, with evidence that it shapes plant population genetics and biodiversity patterns across environmental gradients [24, 25, 26, 27, 28]. These principles, established in natural systems, provide a conceptual foundation for managing PSF in agriculture [29]. In agriculture, however, PSF applications have remained narrow, focused largely on pathogen accumulation and its mitigation through crop rotation [23, 30]. This overlooks the broader spectrum of PSF, which includes beneficial relationships with mutualistic microbes and decomposers critical for nutrient cycling, stress resilience, and soil health [28, 29, 31, 32]. Moreover, selective breeding for yield has proceeded without consideration of these feedback dynamics, potentially altering key interactions with soil biota relative to wild relatives [33, 34, 35]. Consequently, there is an urgent need to understand how to harness PSF for sustainable agriculture, specifically to reduce fertilizer inputs, improve resource‑use efficiency, and suppress pests and diseases [28, 32].

**Figure 1:**
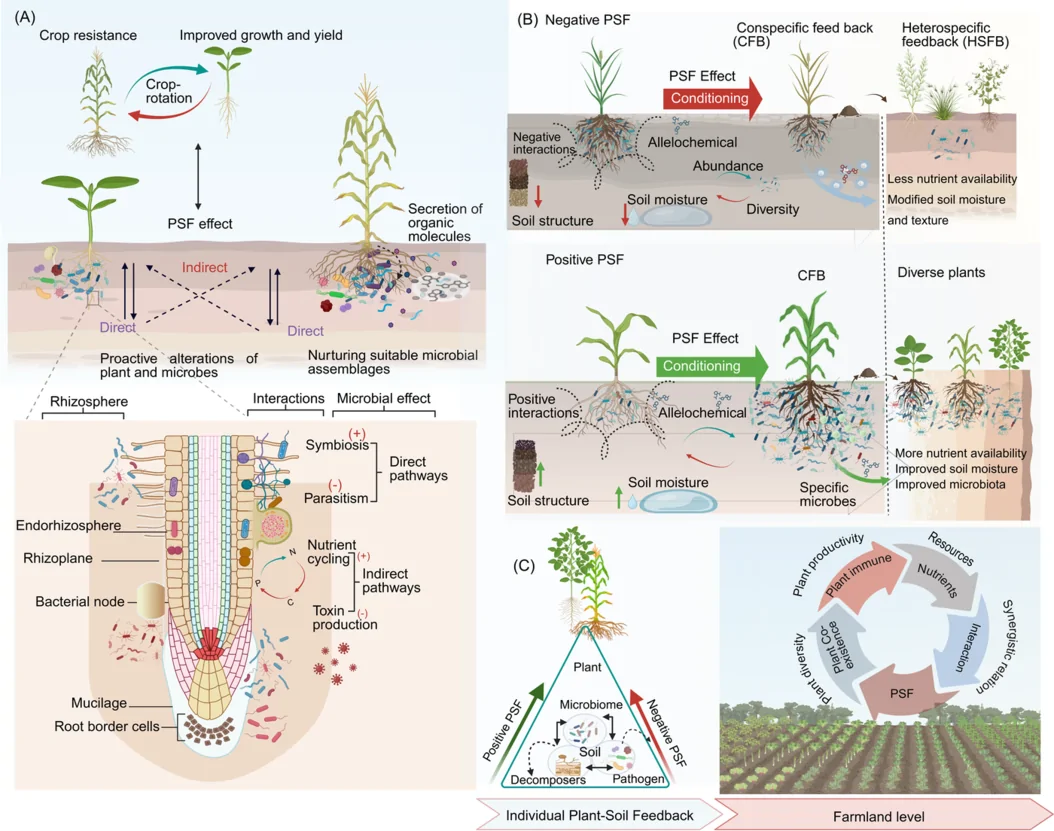
Multiscale depiction of plant‐soil feedback (PSF) dynamics influencing crop resistance, growth, and yield at individual to farmland levels. (A) Illustration of positive PSF mechanisms promoting crop resistance and enhanced growth through direct and indirect plant–microbe interactions. Crop rotation facilitates proactive alterations in the rhizosphere, including the endorhizosphere, rhizoplane, bacterial nodes, mucilage secretion, and root border cells. These foster symbiotic and parasitic interactions, alongside microbial effects such as nutrient cycling (N, P, C) and toxin production, leading to nurturing of suitable microbial assemblages and overall improved plant performance. (B) Comparative analysis of negative and positive PSF under conspecific (CFB) and heterospecific (HSFB) feedbacks. In negative PSF scenarios, conditioning phases involve no interactions, allelochemical interference, or pathogenic influences, resulting in degraded soil structure, reduced nutrient availability, diminished microbial diversity, and modified soil moisture and texture. Conversely, positive PSF exhibits enhanced interactions, diversity, beneficial allelochemicals, and specific microbes, yielding improved nutrient cycling, soil structure, moisture retention, and microbial productivity. (C) Hierarchical and cyclical representation of PSF at the individual plant level, portrayed as a pyramid with positive PSF driven by resilient microbiomes counteracting drought, soil degradation, and pathogens, contrasted against negative PSF at the base. This integrates into a farmland‐scale cycle encompassing plant immune responses, nutrient uptake, plant–microbe co‐evolution, strategic PSF modulation, and enhanced plant resistance, ultimately optimizing agricultural productivity across landscapes. C, carbon; CFB, conspecific feedback; HSFB, heterospecific feedbacks; N, nitrogen; P, phosphorus; PSF, plant‐soil feedback.

Although ecological research has elucidated the mechanistic basis of PSF, its deliberate management as a tool for designing resilient farming systems remains underdeveloped. Traditional practices such as crop rotation implicitly apply PSF principles, but economic pressures have shortened rotations and increased reliance on chemical inputs that can harm beneficial soil organisms [12, 36]. A modern, tool‐based view of PSF seeks to engineer this feedback actively. This involves not only suppressing negative feedbacks from pathogens but, more importantly, fostering positive feedbacks by soil microbiomes that enhance plant growth, nutrient use efficiency, and abiotic stress tolerance [37]. Bridging this gap between ecological theory and agricultural practice is essential for reducing input dependency and enhancing system sustainability [37, 38].

A key but often overlooked determinant of PSF strength and direction is the spatial architecture of microbial interactions, not merely which microbial members are present. This review synthesizes current knowledge on microbiome‐mediated PSF with an explicit focus on its application as a tool for resilient agriculture. We argue that the recent meta‐omics revolution is the enabling technology that transforms PSF from a conceptual model into a diagnosable, quantifiable, and agriculturally deployable force. Simultaneous profiling of microbial community potential (metagenomics), active functional expression (metatranscriptomics), protein‐level metabolic output (metaproteomics), and metabolomics provides, for the first time, a multi‐dimensional empirical window into the plant–microbe–soil nexus that PSF theory describes. For example, genome‐centric culture‐enriched metagenomics now resolves the functional stratification of soil communities across environmental gradients, linking specific genomic potential to temperature‐driven reassembly [37, 38]. Without these tools, PSF remained largely observational; with them, it becomes tractable for management.

Our synthesis is structured around three strategic pillars for harnessing positive PSF. Primarily, (i) plant genetics and root exudates not only recruit beneficial microbiomes and develop effective microbial inoculants, but also serve as a first pillar of framework “plant partner engineering.” This can be further harnessed by (ii) second pillar soil habitat management, which includes agronomic practices (diversification, organic amendments, reduced tillage) to shape microbial habitat and resource availability. Finally, defense priming and enhanced nutrient cycling feedbacks come under the third pillar of targeted biocontrol and decomposer optimization to suppress soil‐borne pathogens through decomposer communities. These pillars map directly onto the diagnostic framework we propose: farming practices, resource availability and efficiency, and microbial habitat conditions serve as the core diagnostic axes for assessing and intervening in PSF dynamics. Multi‐omics tools provide the empirical foundation for diagnosing soil feedback states, monitoring intervention outcomes, and building the mechanistic‐predictive models required for precision PSF management. Our aim is to translate mechanistic understanding of plant–microbe–soil interactions into practical strategies, advocating for an ecological approach to agriculture that ensures long‐term productivity and environmental sustainability.

## 2 DYNAMICS OF PSF: FROM DIRECTION TO EFFECT

A clear classification of PSF types is a prerequisite for their purposeful application in agriculture. PSF is distinguished along two principal axes: the nature of the effect (positive or negative) and the level of interaction specificity (direct or indirect) [39]. Direct PSF occurs when a plant modifies its own soil environment and subsequently experiences altered performance as a consequence (Figure 1A−C) [24, 28]. Direct feedback can be negative, typically driven by the accumulation of host‑specific pathogens or autotoxic allelochemicals (Figure 1A) [40], or positive, often resulting from enrichment of mutualists such as arbuscular mycorrhizal fungi (Figure 1B) [41, 42, 43]. In agricultural systems, negative direct PSF explains the well‐known yield decline under continuous monocropping (Figure 1A,B). Indirect PSF occurs when one plant species alters soil biotic or abiotic properties in a way that differentially affects a different, subsequently grown species (Figure 1B) [44]. This mechanism is the ecological foundation of crop rotation and intercropping: a preceding crop conditions the soil to the advantage of the following crop, either by suppressing pathogens that afflict the subsequent species (negative indirect PSF) or by enriching mutualists that benefit it (positive indirect PSF) [41, 45, 46].

The strength and outcome of this feedback are not fixed but are critically modified by plant–plant interactions in the community (Figure 1A,B) [20, 40]. Conspecific neighbors (same species) amplify direct PSF effects by promoting the accumulation of shared, species‑specific soil biota via rhizodeposition. Heterospecific neighbors (different species), in contrast, dilute these effects by introducing distinct microbial communities (Figure 1B) [39]. This neighbor‐mediated modulation has profound implications for microbiome assembly and function. Experimental evidence indicates that transmission of microbiomes between conspecific hosts drives microbial community specialization, potentially intensifying either positive or negative host‑specific feedbacks [47]. Transmission between heterospecific hosts, by contrast, favors microbial generalism, which can stabilize community function and suppress host‑specific pathogens [47, 48]. This mechanistic insight explains why diverse plant communities often exhibit positive, community‑level feedbacks and overyielding, whereas monocultures are prone to negative feedback cycles. Negative indirect feedbacks are often documented in studies related to invasive plant species (Figure 1B) [40].

Originally developed to explain coexistence in nature, PSF theory posits that these feedbacks generate critical linkages between genetic diversity (within a species) and species diversity (within a community) [27, 39]. Negative PSF, where fitness is reduced in self‐conditioned soil, acts as a cornerstone for maintaining both levels of diversity by preventing any single genotype or species from dominating [49]. The valency of these feedbacks determines the synergy between diversity levels. High genetic diversity in a focal species leads to more negative (or less positive) interspecific PSF, for example, by diluting host‑specific pathogens. More negative interspecific PSF promotes species coexistence, thereby maintaining higher species diversity. In turn, high species diversity leads to more negative (or less positive) intraspecific PSF for a focal species, which promotes genotype coexistence and sustains higher genetic diversity. The net effect is a positive feedback loop where diversity at one level reinforces the other [27, 50]. High genetic diversity dilutes conspecific pathogens, weakening negative interspecific PSF (making it more positive or less negative). Weaker negative interspecific PSF reduces the force for species coexistence, leading to lower species diversity. Conversely, high species diversity dilutes genotype‐specific pathogens, weakening negative intraspecific PSF. Weaker negative intraspecific PSF reduces genotype coexistence, leading to lower genetic diversity. The net effect is a negative relationship where high diversity at one level undermines the mechanisms maintaining diversity at the other level [27, 49, 50]. Therefore, the strategic imperative for resilient agriculture is twofold: (i) mitigate negative direct PSF by breaking conspecific cycles, and (ii) promote positive indirect PSF by designing diversified systems that harness heterospecific effects to build disease‐suppressive soil microbiomes [51]. Achieving this goal requires targeted manipulation of the rhizosphere microbiome via plant genetics and the regulation of root exudation.

## 3 MICROBIAL MECHANISMS AND PATHWAYS DRIVING PSF VIA HOST GENETICS AND ROOT EXUDATE COMPOSITION

### Host genetics dynamics harness the positive PSF

Host genetics influence microbiome assembly through a hierarchy of mechanisms: receptor‐mediated signaling filters microbial partners [52]; transcriptional regulation of metabolic pathways produces specific recruitment cues; and developmental and transporter genes construct the physical and chemical landscape of the rhizosphere [53, 54]. Rhizosphere microbiome assembly is not a passive process; it is governed by plant genetic programs that regulate root development, immune signaling, and metabolic output (Figure 2A) [53]. These programs operate through specific molecular pathways that shape microbial community composition and function, ultimately determining the direction and strength of PSF [55, 56]. Receptor‑mediated signaling acts as an initial filter for microbial partners [57]. The FERONIA receptor kinase, for example, is a central regulator of root growth and stress responses [57]. Mutations in *FERONIA* receptor kinase alter pathogen‐induced reactive oxygen species (ROS) burst and selectively enrich for beneficial *Pseudomonas* strains in the rhizosphere [58]. Many of these recruited *Pseudomonas* carry biosynthetic genes for antimicrobial compounds such as phenazines or the resistance‐priming signal 2,4‐diacetylphloroglucinol (DAPG) [59]. Thus, a single signaling component can shift community composition toward a protective phenotype, creating a positive, pathogen‐suppressive PSF. Similarly, the Nod factor signaling pathway in legumes, involving receptors like *NFR1‐NFR5* [60], is essential for the precise recognition of compatible rhizobia, ensuring the formation of nitrogen‐fixing nodules while excluding ineffective strains (Figure 2A,B) [61, 62]. These signaling cascades frequently integrate environmental cues, allowing plants to dynamically adjust microbial recruitment in response to nutrient status or pathogen attack [63].

**Figure 2:**
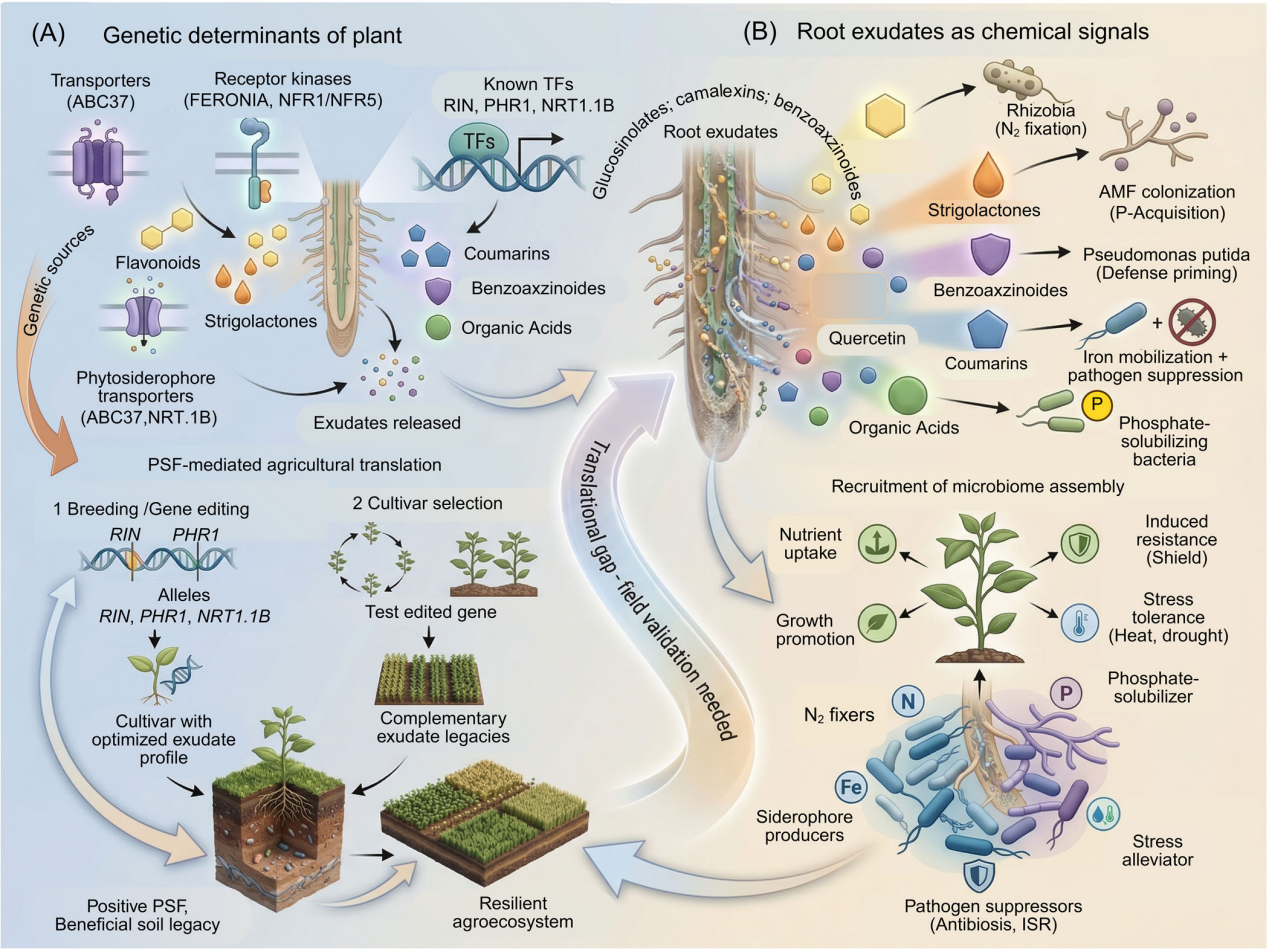
Genetic regulation of root exudates, microbiome recruitment, and translation into plant‐soil feedback (PSF) mediated agriculture. (A) Genetic determinants of plant: Plant genetic pathways, including transcription factors (TFs) (e.g., RIN, PHR1, NRT1.1B), receptor kinases (e.g., FERONIA, NFR1/NFR5), and transporters (e.g., ABC37), regulate the synthesis and release of specific root exudates. Signal transduction pathways, such as those involving NRT1.1B‐like modules and specific genetic loci (e.g., SHORTROOT‐SCARECROW), mediate these interactions, drawing from a wild genotype pool to target genetic breeding objectives. This results in resilient crop varieties with a wider gene pool, associated with diverse microbes and synthetic communities (SynCom), and enhanced tolerance to biotic and abiotic stresses following PSF. PSF‐mediated agricultural translation (Bottom left): Understanding these genetic and chemical interactions can be leveraged for agricultural improvement. Through gene editing or breeding of specific alleles (e.g., *RIN*, *PHR1*, *NRT1.1B*) and strategic cultivar selection, crops can be developed with optimized exudate profiles. A translational gap indicates the ongoing need for field validation of these optimized traits. (B) Root exudates as chemical signals: The released exudate cloud contains distinct secondary metabolites that recruit specific beneficial microbes. For example, flavonoids recruit Rhizobia for nitrogen fixation; strigolactones promote arbuscular mycorrhizal fungi (AMF) colonization for phosphorus acquisition; benzoxazinoids attract *Pseudomonas putida* for defense priming; coumarins facilitate iron mobilization and pathogen suppression; and organic acids attract phosphate‐solubilizing bacteria. Recruitment of microbiome assembly (Bottom Right): The assembled beneficial microbiome provides multifaceted support to the plant, including enhanced nutrient uptake (N, P, Fe), growth promotion, ISR (shielding against pathogens), and alleviation of abiotic stresses like heat and drought. All of these foster complementary exudate legacies and positive PSF, ultimately designing a resilient agroecosystem. ISR, induced systemic resistance; N, nitrogen; P, phosphorus; Fe, ferrous (iron).

Plant genomes encode the biosynthetic pathways for root exudates that function as microbial attractants, stimulants, or inhibitors [64]. Natural variation in these pathways underpins genotypic differences in microbiome assembly. In maize, natural variation in flavonoid biosynthesis genes leads to distinct exudate profiles of compounds like luteolin [65, 66]. These flavonoids act as structural precursors and signals, stimulating specific microbial consortia (e.g., Oxalobacteraceae) that contribute to plant health through hormone modulation or niche competition [67, 68]. In addition, these specific flavonoid profiles act as chemical cues that facilitate colonization by these growth‐promoting bacteria [53, 65, 68]. In tomato, the Ripening‐Inhibitor (*RIN*) transcription factor directly regulates the production of root exudates that enrich for antibiotic‐producing *Streptomyces*, linking a master developmental regulator to microbiome‐mediated disease resistance (Figure 2A) [69]. Under nutrient deficiency, plants activate starvation responses that alter exudation. The phosphate‐starvation response (PSR) network, coordinated by transcription factors like *PHR1*, induces the secretion of (e.g., citrate, malate) and flavonoids that recruit phosphate‐solubilizing bacteria (PSB) [70]. These exudates do more than solubilize minerals; they serve as selective carbon sources and signals for PSB and fungi. This establishes a nutritional feedback module: plant sensing of P‐limitation triggers a microbial recruitment strategy that directly alleviates the nutrient stress, creating a positive PSF for nutrient acquisition. Under iron stress, the *bHLH* transcription factor FER‐LIKE IRON DEFICIENCY‐INDUCED TRANSCRIPTION FACTOR (FIT) upregulates the biosynthesis of coumarins (e.g., scopoletin, fraxetin), which are secreted via transporters like *ABCG37/PDR9* [71]. Coumarins perform dual functions: they chemically mobilize insoluble iron, and they selectively inhibit fungal pathogens while enriching for bacteria that produce iron‑chelating siderophores [72, 73]. This module converts a plant stress state into a microbiome‑mediated solution for both nutrient limitation and disease, steering a potential negative PSF toward a positive outcome [74].

Root architecture and transport systems physically shape the microbial habitat. The SHORTROOT‑SCARECROW (SHR‑SCR) module controls radial root patterning and meristem maintenance, thereby determining exudation zones and physical niches for microbial colonization (Figure 2A,B) [75]. Transporters that release or retain root metabolites are equally critical. For example, in sorghum, a mutant lacking a phytosiderophore transporter fails to release iron‐chelating compounds, which unexpectedly enriches for drought‐tolerant Actinobacteria, revealing how a single transporter mutation can restructure the microbiome for stress adaptation [76]. Similarly, nitrate transporter *NRT1.1B* in rice influences the abundance of nitrogen‐cycling bacteria in the rhizosphere, connecting host nitrogen uptake genetics to microbial nitrification processes (Figure 2A,B) [77]. These specific genetic loci and their products, such as those involved in the SHR‐SCR module [75], nitrate transporters like *NRT1.1B* [77], and the phosphate starvation response network [70] have been shown to shape the microbiome directly, but the studies lack their functional implementation in breeding program to harness positive PSF [78].

The identification of these molecular pathways provides actionable targets for microbiome‐assisted breeding [79]. The goal is to pyramid favorable alleles to create cultivars that proactively construct resilient soil legacies. Polymorphisms in key genes (e.g., *RIN* in tomato, *PHR1*, biosynthetic enzymes for coumarins/flavonoids) are being translated into molecular markers for marker‐assisted selection [80]. This allows breeders to screen for optimal microbiome recruitment traits without extensive microbial phenotyping. While many of these loci remain powerful research tools for elucidating plant–microbe dialogue, several are transitioning into targets for applied breeding. For example, genome‐wide association studies (GWAS)‐derived rice alleles [81], that promote nutrient‐efficient microbial recruitment, are currently being introgressed into elite lines to enhance crop resilience [82].

These mechanistic insights provide a roadmap for molecular breeding and gene editing aimed at optimizing PSF [83]. Rather than selecting for yield alone, breeders can target alleles of the aforementioned genes (e.g., *FERONIA*, *RIN*, *PHR1*, *NRT1.1B*) that favor the recruitment of beneficial microbiomes (Figure 2A,B) [83]. The emerging strategy of holobiont breeding seeks to improve crop performance by treating the plant and its microbiome as a co‐evolved unit. This involves screening diverse germplasm for microbiome‐associated traits and leveraging genetic markers linked to favorable microbial recruitment [33, 84]. The identification of these molecular pathways, receptor‐mediated microbiome filtering, transcriptional regulation of exudate chemistry, and developmental control of root architecture, provides a mechanistic roadmap for plant breeding aimed at optimizing PSF; the strategies by which these insights are translated into cultivar improvement are the subject of a section “integrating PSF toolbox via complex interplay of microbiome‐assisted breeding, holobiont, and rewilding strategies.”

### Root exudates: Chemical signals for microbiome recruitment and mitigation of negative PSF

Root exudates constitute the primary biochemical interface between plants and soil, forming a complex metabolome that structures the rhizosphere microbiome [85, 86]. This secretion is a dynamic, regulated process integral to PSF, where plants actively shape their soil environment to enhance fitness (Figure 2A,B) [84, 86]. Far beyond a simple carbon subsidy, exudates function as precise chemical signals that selectively recruit microbial consortia, suppress pathogens, and coordinate adaptive responses to abiotic stress (Figure 2A,B) [85, 86]. Harnessing this bidirectional chemical dialogue is therefore a foundational strategy for engineering resilient rhizospheres.

Root exudates comprise a diverse array of primary metabolites (e.g., sugars, amino acids, organic acids) and specialized secondary metabolites (e.g., flavonoids, strigolactones, coumarins, benzoxazinoids) (Figure 2A,B) [87, 88]. Primary metabolites serve as labile carbon sources that fuel microbial growth and modulate soil nutrient and carbon dynamics via the rhizosphere priming effect [89]. Rhizosphere microbes actively modify and transform these plant‐derived compounds, establishing a complex bidirectional signaling coordination that profoundly influences PSF (Figure 2A,B) [90]. In this chemical dialogue, bacterial sigma factors perceive host‐derived and environmental cues, directly coupling stress perception to transcriptional reprogramming of virulence and symbiosis traits [87, 88, 91]. In a microbe‐oriented domain, they can degrade or detoxify plant‐produced allelochemicals, thereby mitigating their inhibitory effects on plant growth or shaping the competitive landscape within the rhizosphere, and production of secondary metabolites and oxidative mineralization play a regulatory role (Figure 2A,B). The spatial patterning of exudation, visualized using bioreporter strains (*Rhizobium* reporter strains), ensures precise microbial colonization both on the root surface and within internal vascular tissues [91]. Exudate composition is highly responsive to plant developmental stage, nutritional status, and biotic challenges, rendering the rhizosphere a dynamic reflection of plant physiology [88, 92]. This plasticity enables real‐time modulation of microbial recruitment. Under nitrogen limitation, for instance, legumes increase flavonoid exudation to attract symbiotic rhizobia; under phosphorus deficiency, they release organic acids (citrate, malate) that solubilize mineral phosphates and recruit phosphate‐mobilizing bacteria [92, 93, 94].

Certain microbial communities can convert plant exudates into novel signaling molecules that further influence plant gene expression or defense responses [95]. Flavonoids secreted by host roots bind to bacterial *nodD* proteins in compatible rhizobia, initiating nodulation gene expression and establishing nitrogen‑fixing symbiosis (Figure 2B) [60, 95]. Legume‐derived flavonoids are not only perceived by rhizobia but are also taken up and converted by bacterial enzymes (e.g., Nod‐induced O‐methyltransferases) into methylated derivatives that function as stronger or longer‐lasting *Nod* gene inducers [96]. This microbial processing amplifies the symbiotic signal and fine‐tunes nodulation timing. In addition, strigolactones exuded under phosphorus deficiency not only trigger arbuscular mycorrhizal branching but are also enzymatically converted by rhizobacteria into antimicrobial aglycones, transforming a symbiotic signal into a localized toxin (Figure 2B) [97, 98]. A similar activation occurs with benzoxazinoids in maize; these compounds shape a jasmonate‐priming microbiome that enhances systemic resistance against herbivores [99]. In both cases, specialized microbes like *Pseudomonas putida* utilize glycosyl hydrolases liberate toxic aglycone forms from their glucoside precursors, a process that selectively enriches beneficial, tolerant taxa while inhibiting susceptible pathogens [98, 99, 100]. Meanwhile, *Pseudomonas*‐produced DAPG, synthesized from plant carbon substrates, re‐enters the plant as an elicitor of jasmonate/ethylene‐mediated induced systemic resistance, completing a loop from plant carbon to microbial antibiotic to systemic plant defense [59, 100]. Analogous immune priming through specific microbial metabolites is well‐established in animal systems, where *Akkermansia muciniphila*‐derived γ‐aminobutyric acid suppresses nuclear factor kappa B inflammatory signaling to boost antiviral resistance [101]. This illustrates a conserved principle, that a single microbial metabolite can rewire host immunity, supporting the translational potential of metabolite‐based PSF engineering in crops. Coumarins secreted by *Arabidopsis* (e.g., scopoletin, fraxetin) perform dual functions, mobilizing ferric iron and exerting antimicrobial activity. This selectivity favors iron‐efficient mutualists like *Pseudomonas simiae* WCS417 while suppressing pathogens [102]. *P. simiae* further metabolizes coumarins into simpler phenolic compounds that can act as additional iron‐chelating signals, creating a positive feedback loop that reinforces its own rhizosphere dominance [72, 73, 103]. In addition, root exudates secreted along the root axis are even under plants' precise spatial control creating micro‐niches for microbes. Coumarins, for instance, are secreted primarily from root tips under iron deficiency to mobilize ferric iron, which later blends with different metabolite in mature root zones to manage microbial colonization [73]. This compartmentalization enables one root system to simultaneously handle nutrient uptake, symbiotic relationships, and defense mechanisms through localized chemical signals [104, 105].

Plants also deploy systemic chemical signals. Camalexin, a phytoalexin produced in aerial tissues in response to pathogen attack, is translocated to roots and exuded into the rhizosphere, where it selectively inhibits pathogens such as *Burkholderia glumae* while exerting neutral or beneficial effects on tolerant strains like *Pseudomonas* sp. CH267 (Figure 2B) [74, 106]. Tolerant rhizobacteria can detoxify or repurpose camalexin‐derived metabolites, illustrating how microbial metabolism modifies plant‐derived defense compounds [107]. Cucumber plants challenged with *Fusarium oxysporum* exude threonic acid and lysine, recruiting protective *Bacillus* and *Sphingomonas* species that enhance local ROS production and resistance [108, 109]. Local infection of one root system elevates tryptophan and reduces raffinose in distal roots, favoring colonization by *Bacillus amyloliquefaciens*, while *Bacillus* converts tryptophan into the auxin indole‐3‐acetic acid (IAA), which in turn modifies root exudation patterns and reinforces the recruitment of beneficial consortia. This suppresses the pathogen and reconfigures the microbiome toward beneficial associations evidenced by *Azospirillum*, *Rhizobium*, and *Pseudomonas* species [110, 111, 112]. In addition, this feedback also extends to lateral root architecture, increasing rhizosphere surface area and exudate output, forming a self‐amplifying positive PSF loop [113].

Additionally, tea seedlings infected with *Pseudopestalotiopsis camelliae sinensis*, the causal agent of gray blight, significantly alter rhizosphere communities to combat the pathogen [114]. Bacteria possessing 1‐aminocyclopropane‐1‐carboxylate (ACC) deaminase degrade 1‐aminocyclopropane‐1‐carboxylic acid, the ethylene precursor released in elevated amounts by stressed plants, into α‐ketobutyrate [115]. This process diminishes root ethylene accumulation, alleviates growth inhibition, and maintains the carbon flow that supports mutualistic relationships, which would otherwise be disrupted under stress [115]. Microbially modified exudates are not merely local signals; they can be re‐absorbed by roots and trigger systemic responses. For example, the bacterial conversion of root‐secreted l‐malate into the quorum‐sensing signal 3‐oxo‐C12‐HSL by *Pseudomonas aeruginosa* alters root transcriptomes, suppressing defense genes in a manner that facilitates colonization, a phenomenon termed metabolic eavesdropping [116].

These examples illustrate the sophistication of chemical crosstalk, where distinct metabolites summon specific microbial functional guilds (Table 1). Plants can attract beneficial microbes to specific root zones (e.g., root tips) while excluding pathogens from others (e.g., root‐shoot junctions), demonstrating precise spatial control over feedback dynamics (Figure 2A,B) [104, 105]. This reprogramming frequently encourages the formation of a defensive microbiota, which can minimize the harmful repercussions of subsequent attacks by the same aggressor, a phenomenon known as negative feedback mitigation [138]. Such signaling pathways represent co‑evolutionary adaptations that facilitate protective microbial recruitment, underscoring the centrality of root‑exudate‑based PSF in plant health (Figure 2A,B) [20, 28, 139, 140]. Consequently, focused manipulation or genetic engineering of specialized metabolite pathways offers a promising approach to attracting and sustaining specific beneficial bacteria that proactively promotes positive soil legacies [140].

**Table 1:** Agricultural practices shape plant‐soil feedbacks (PSF) by differentially influencing soil microbial functional groups.

Category	Practice	Mechanism	Microbial guild impact	Net PSF	Refs
Conventional and High‐Input	Monoculture	Pathogen buildup through continuous cropping	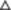 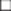 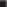 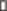 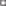 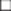	**Negative**	[117, 118]
	Excessive Fertilizer	Suppression of mutualists; increased pathogen activity	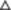 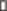 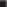 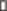 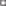 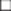	**Negative**	[119, 120, 121, 122]
	Pesticide Overuse	Broad‐spectrum elimination of soil biota	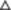 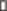 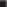 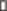 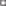 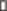	**Uncertain**	[7, 12, 123, 124]
Agroecological and Diversified Systems	Rotation	Increased biodiversity; pathogen disruption	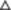 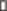 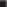 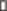 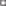 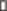	**Positive**	[30, 125]
	Intercropping	Beneficial recruitment; pathogen dilution	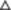 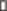 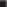 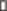 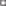 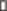	**Positive**	[117, 126]
	Manure/Compost	Higher diversity; enhanced suppressive microbiome	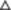 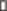 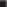 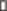 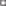 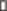	**Positive**	[17, 127, 128]
	Reduced/No‐Till	Organic matter accumulation; stable beneficial networks	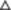 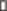 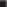 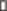 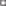 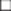	**Positive**	[129, 130, 131]
	Cover Cropping	Biodiversity introduction; beneficial recruitment	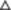 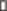 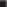 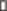 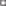 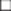	**Positive**	[108, 132, 133, 134, 135, 136]
Targeted Biological Strategies	Nutrient‐Efficient Genotype	Recruits' nutrient‐mobilizing mutualists	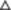 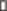 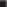 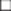 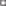 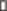	**Positive**	[77, 137]
	Pathogen‐Control Genotype	Enriches protective/suppressive microbes	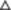 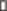 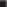 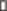 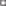 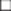	**Positive**	[58, 69]

*Note*: 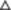 Symbiont (arbuscular mycorrhizal fungal (AMF), Rhizobia); 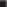 Pathogen/Pest; 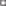 Decomposer/Saprophyte; 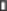 increased/promoted; 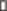 decreased/suppressed; 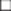 uncertain or context‐dependent. Overall expected influence of the practice on crop performance, derived from guild‐level shifts.

Identifying key exudate signals allows for two complementary approaches. First, it facilitates the development of microbial communities, such as stress‐tolerant strains of *Pseudomonas* or *Streptomyces* [141, 142], suitable for specific crops and capable of promoting beneficial interactions. Efforts to isolate microorganisms with defined functions, including mutualists such as rhizobia and arbuscular mycorrhizal fungi and stress‑alleviating strains for drought and salinity, are ongoing with the goal of developing tailored inoculants (Figure 2A,B) [141, 142] for use as tailored microbial inoculants [143]. Genomic prospecting of extreme environments continues to expand this toolkit; for instance, polyphasic taxonomy of desert *Kineococcus* isolates has uncovered nine novel species carrying plant growth‐promotion genes [139, 144] underscoring the value of underexplored habitats for discovering next‐generation inoculants. Similarly, novel *Nocardioides* species with proteolytic and ureolytic capabilities have been isolated from desert rhizosphere soils [140, 145], indicating that arid‐adapted rhizospheres harbor a functionally diverse reservoir of nutrient‐cycling strains. On the other hand, exudate chemistry provides a scientific basis for designing strategic cropping systems [141, 142]. Together, these plant‑centric mechanisms demonstrate how genetic and metabolic traits can be harnessed to recruit beneficial microbiomes, which is one part of the PSF equation. The other half is mediated by a distinct but equally critical guild: soil decomposers, and soil health and crop productivity depend equally on their activities.

## 4 THE DUAL ROLE OF SOIL DECOMPOSERS IN PSF

Soil decomposers are central mediators of PSF. Through the breakdown of plant‑derived organic matter (litter and root exudates), they regulate the transformation of carbon and nutrients from plant tissues into bioavailable forms, thereby creating feedback loops that can be either positive or negative for plant growth and soil health (Figure 3A). The rate and outcome of this decomposition are largely governed by the quality of plant inputs, such as litter chemistry and exudate composition [145]. These organisms, including fungi, bacteria, and invertebrates, regulate the critical transition of carbon and nutrients from plant tissue back into bioavailable forms, thereby creating feedback loops that can be either positive or negative for plant growth and soil health (Figure 3A). A well‑documented manifestation of positive PSF mediated by decomposers is home‑field advantage: decomposer communities specialize in breaking down litter from the local plant species, accelerating nutrient release, and benefiting the resident vegetation [146, 147, 148]. Conversely, decomposer activity can also drive negative PSF (Figure 3A). Priming, the stimulation of microbial decomposition of stable soil organic matter by fresh root exudates, may cause a net loss of soil carbon, thereby undermining long‑term fertility, water retention, and carbon sequestration [149, 150]. Decomposition can also release biochemical legacies, allelopathic compounds, or extracellular DNA, that inhibit the growth of subsequent plants (Figure 3A) [151, 152, 153].

**Figure 3:**
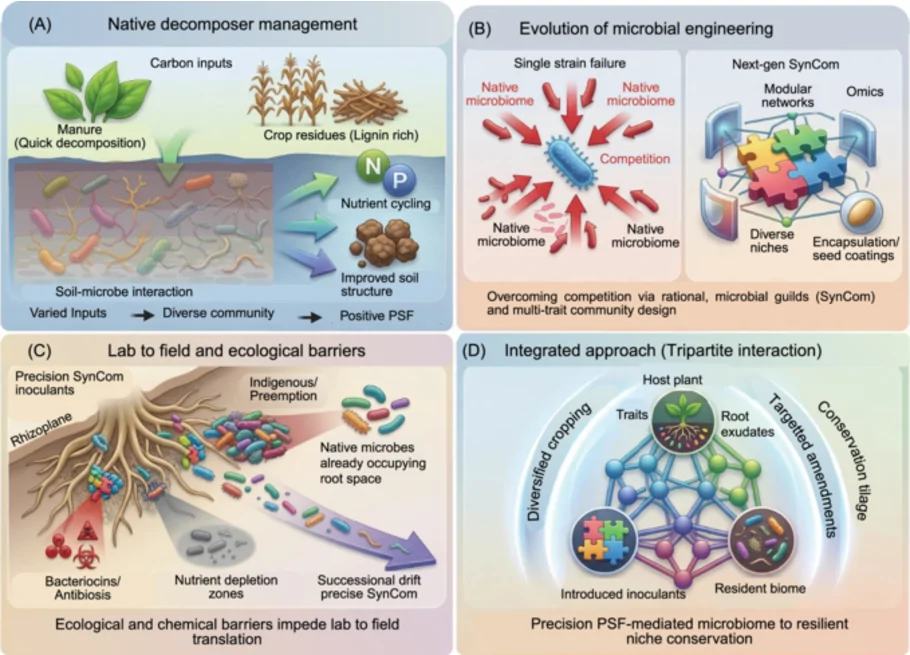
Strategies, challenges, and integrated approaches in microbial engineering for positive plant‐soil feedback (PSF). (A) Native decomposer management: Highlights how varied carbon inputs—ranging from quick‐decomposing manure to lignin‐rich crop residues—stimulate diverse soil microbial interactions. This diversity enhances nutrient cycling (e.g., nitrogen and phosphorus), improves soil structure, and drives positive PSF. (B) Evolution of microbial engineering: Contrasts traditional and modern inoculation strategies. Single‐strain failure occurs when individual introduced strains are outcompeted by the established native microbiome. Next‐generation synthetic communities (Next‐Gen SynComs) overcome this competition by utilizing multi‐trait community design, omics data, modular networks to occupy diverse niches, and advanced delivery methods like encapsulation or seed coatings. (C) Lab to field and ecological barriers. It illustrates the hurdles in translating lab‐developed inoculants to real‐world applications. Precision SynCom faces significant ecological and chemical barriers at the root interface (rhizoplane), including preemption by indigenous microbes already occupying root space, competition in nutrient depletion zones, antibiosis (e.g., bacteriocins), and successional drift over time. (D) Integrated approach (Tripartite interaction) proposes a holistic framework for precision PSF‐mediated microbiome management. Achieving resilient niche conservation requires synergizing three core components: the host plant (leveraging specific traits and root exudates), introduced inoculants, and the resident biome. This tripartite network is stabilized and supported by broader agroecological management practices, including diversified cropping, targeted amendments, and conservation tillage.

Given this dual capacity, the strategic management of decomposer communities becomes a key tool for steering PSF toward positive outcomes. Although directly engineering these complex communities is currently unfeasible, agronomic practices can shape their function by manipulating the quality and quantity of carbon inputs [154]. Using a range of organic amendments, from quick‐decomposing green manures to slower, lignin‐rich crop residues, promotes a diverse community of decomposers [155, 156]. This diversity improves nutrient cycling and soil structure (Figure 3A). This principle extends to plant diversity within cropping systems; varied litter and exudate profiles from cover crop mixtures or intercropping support more complex decomposer networks, which can buffer against pathogen build‐up and improve nutrient‐use efficient community (Figure 3A) [12, 154, 155, 156]. When coupled with conservation practices such as reduced tillage that preserve decomposer habitats, this approach shifts the objective from merely adding organic matter to actively cultivating a decomposer community that generates resilience‑building feedbacks (Figure 3A).

Translating these mechanistic insights into reliable farm‑scale tools requires overcoming a substantial lab‐field gap [78]. A primary obstacle is the poor establishment of beneficial microbes introduced as inoculants. Traditional single‑strain inoculants often fail to persist in field soils because they face intense competition from resident communities (Figure 3B) [157]. This limitation has spurred the development of next‑generation solutions, including advanced seed coatings and encapsulation technologies that protect microbial cells, as well as the rational design of synthetic microbial communities (SynComs) (Figure 3). The rational design of SynComs represents the most direct application of interaction network principles to PSF engineering. Unlike single‐strain inoculants, which compete against the full resident microbiome, SynComs designed around ecologically coherent guild structures can occupy multiple functional niches simultaneously, reproduce the modular network topology of naturally suppressive soils, and generate emergent functions, including cascading antibiosis, cross‐fed metabolites, competitive exclusion via niche pre‐emption, that no single strain can deliver (Figure 3C) [54, 143, 158]. These engineered consortia informed by multi‐omics and machine learning, aim to perform complex functions like targeted pathogen suppression or nutrient mobilization [158, 159]. These SynComs show promises for delivering agronomically valuable traits, including enhanced nitrogen and phosphorus availability, improved drought and salinity tolerance, and pathogen suppression (Figure 3B) [160, 161]. Importantly, the scientific community has recognized these translational challenges, and coordinated efforts are underway to improve the reproducibility and transferability of SynCom‑based approaches. These include the development of standardized frameworks for the design, reporting, and benchmarking of microbiome experiments [158, 159] (Figure 3).

Despite their robust design, the field reliability of SynComs is frequently undermined by intense ecological competition and rapid successional drift [162]. In unmanipulated agricultural soils, indigenous communities often preemptively occupy available niches, deploying bacteriocins and creating nutrient‐depletion zones that restrict inoculant establishment to below 10% of applied cells, regardless of dosage (Figure 3C) [78, 163, 164]. This intense competition for rhizoplane niches and shared carbon sources triggers a rapid divergence in member‐strain ratios, destabilizing the SynCom's intended proportions and compromising the functional complementarity derived from multi‐omics modeling [165]. Beyond immediate establishment failures, the deployment of SynComs carries long‐term ecological risks, including the potential for horizontal gene transfer of antibiotic resistance or metabolic traits to resident taxa [166]. Furthermore, the displacement of indigenous species may erode the functional redundancy of the native microbiome in ways that remain undetected on standard agronomic timescales. Acknowledging these hurdles does not diminish the promise of SynComs, but rather defines the research priorities essential for their successful transition from the laboratory to the field [162].

The ultimate success of PSF‐based applications hinges on a mechanistic understanding of the tripartite interactions between the host plant, the introduced inoculants, and the resident soil biome (Figure 3D). Currently, a fundamental barrier to deploying PSF as a predictive tool remains the poor generalizability of findings from controlled environments to the stochasticity of field conditions [167]. The complex interplay between host traits, fluctuating climates, and diverse edaphic variables renders the isolated engineering of soil communities largely unfeasible for broad application (Figure 3D) [167, 168]. Consequently, the field is shifting toward an integrated systems approach that combines diversified cropping, targeted organic amendments, and conservation tillage with precision microbial inputs to harness the full potential of PSF in resilient agriculture (Figure 3D).

## 5 INTEGRATING AGRONOMIC PRACTICES AS A TOOL TO HARNESS POSITIVE PSF

While precision microbiome engineering, including SynComs and exudate manipulation, offers transformative potential, its field‐scale translation is often constrained by ecological stochasticity, competitive exclusion by resident microbiota, and economic scalability [129]. In response to these challenges, broader‐scale agronomic strategies, such as crop diversification, organic amendments, and conservation tillage, provide a robust, habitat‐first approach to steering PSF. Rather than relying on the precarious establishment of individual strains, these top‐down management practices modify the soil physicochemical environment to systematically favor beneficial microbial recruitment and suppressive functions (Table 1 and Figure 4). Consequently, strategic agronomic management represents a foundational pillar for PSF applications, transitioning from isolated microbial inoculants toward a systems‐level engineering of the soil‐crop holobiont.

**Figure 4:**
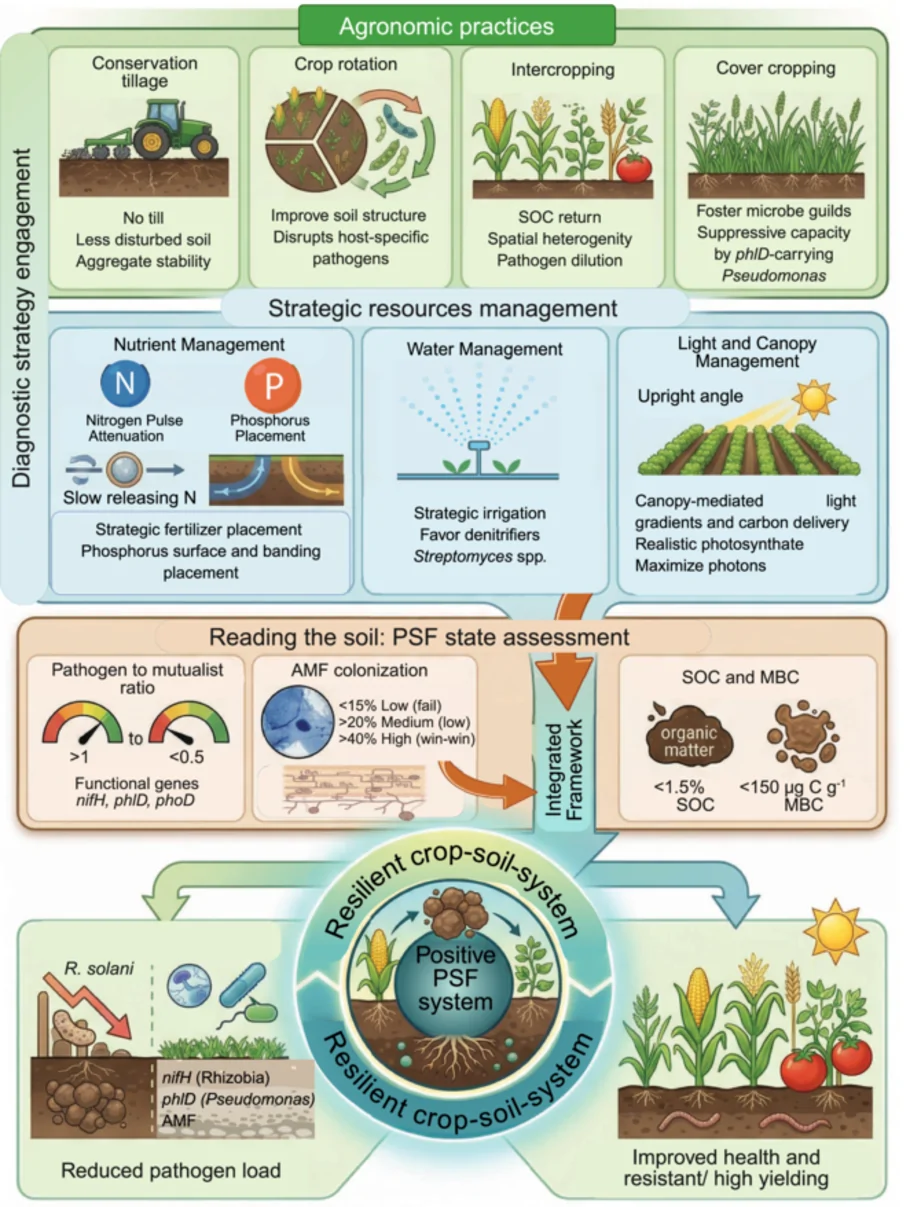
An integrative management framework for operationalizing plant‐soil feedback (PSF) steerage in resilient agroecosystems. The framework depicts a hierarchical, diagnostic‐led strategy for transitioning from pathogen‐conducive to suppressive soil states. Agronomic Practices serve as the structural foundation, utilizing conservation tillage to improve aggregate stability, and strategic crop rotation and intercropping to increase spatial heterogeneity and dilute host‐specific pathogens. Cover cropping is positioned as a primary tool for fostering specific beneficial guilds, such as *phlD*‐carrying *Pseudomonas*. Strategic Resource Management tier identifies water, nutrients, and light as active levers for microbial recruitment. It highlights nitrogen pulse attenuation and targeted phosphorus placement to avoid symbiosis suppression, alongside strategic irrigation and canopy architecture management to optimize the rhizosphere's chemical and radiative environment (*e.g*., favoring *Streptomyces* spp.). PSF state assessment (Reading the soil) is a diagnostic interface that integrates quantitative biological and physicochemical indicators, including the Pathogen‐to‐mutualist (P:M) ratio, functional gene abundance (*nifH*, *phlD*, *phoD*), and arbuscular mycorrhizal fungal (AMF) colonization levels. These metrics inform the integrated framework for state‐contingent interventions. The resilient crop‐soil system. Successful management culminates in a positive PSF system, characterized by a significant reduction in pathogen pressure (e.g., *Rhizoctonia solani*), the enrichment of beneficial functional genes, and improved crop health, resistance, and sustainable yield. MBC, microbial biomass carbon; *nifH*, nitrogenase reductase gene; *phlD*, 2,4‐DAPG biosynthesis gene; *phoD*, alkaline phosphatase gene; SOC, soil organic carbon.

### Conservation tillage: Engineering soil structure and microbial habitat

Practices like no‐till or reduced tillage minimize soil disturbance, strengthening soil physical and biological health (Figure 4) [130]. They boost soil organic carbon, foster microaggregates that protect carbon, and provide habitats for microbial networks [129, 132], by providing a stable environment and, with necromass formation, contribute to a persistent, functional carbon pool [169, 170]. These improvements collectively lead to greater efficiency in water and nutrient use, supporting more sustainable crop production [129, 171]. For example, long‐term studies show that no‐till systems can increase wheat yields while simultaneously increasing SOC and total nitrogen concentrations [172]. This habitat conservation bolsters mutualistic partnerships, including both symbiotic and free‐living nitrogen‐fixing bacteria (e.g., *Azospirillum*, *Pseudomonas*), and can enhance populations of beneficial arthropods and microbially mediated pathogen suppression [130, 173, 174]. Thus, no‐till and reduced tillage enhance PSF primarily by increasing SOC and aggregate stability, thereby protecting the hyphal networks of arbuscular mycorrhizal fungal (AMF) and creating refugia for microbial predators that regulate pathogen populations (Figure 4) [175]. However, the direction and magnitude of PSF effects are strongly modulated by soil texture, climate, and rotation history. A 20‐year meta‐analysis of 106 studies found that no‐till increased SOC by 12.4% in humid temperate climates but only 4.2% in semi‐arid regions, and these gains required at least 5 years to manifest [175, 176]. In a 12‐year maize‐soybean rotation in the U.S. Midwest, no‐till combined with cover cropping increased AMF root colonization by 32%–47% and phosphatase activity by 28%, correlating with a 9% yield increase in low‐phosphorus soils [177, 178, 179].

While symbiotic nitrogen‐fixing bacteria like rhizobia are well‐known for colonizing root nodules [180], non‐symbiotic diazotrophs such as *Azospirillum brasilense* and *Pseudomonas stutzeri* also fix atmospheric nitrogen in the rhizosphere, utilizing root exudates as an energy source [174]. Other free‐living nitrogen‐fixing genera, including *Burkholderia* and *Herbaspirillum*, colonize various plant niches [181, 182, 183], though their specific roles in PSF remain underexplored. The benefits of reduced tillage extend beyond nutrient cycling. By providing a less disturbed habitat, it can bolster populations of beneficial arthropod predators and enhance microbially mediated antagonism against pathogens (Figure 4) [172, 184].

Pathogen carry‑over on retained residues is a well‑documented trade‑off. In continuous no‑till wheat, *Fusarium pseudograminearum* inoculum density increased 3.2‑fold compared to conventional tillage, leading to 17% yield suppression in susceptible cultivars [184, 185, 186]. This risk is mitigated when no‐till is paired with diversified rotations that include non‐host crops (e.g., canola or pulse crops) for at least 2 years. Conservation tillage tends to concentrate plant debris and, consequently, microbial biomass in the top 5 to 15 cm of soil, thereby promoting the survival of pathogens [185]. Conversely, in soils with pH 5.5–6.5 and SOC > 2%, *phlD‐Pseudomonas* populations sustain suppressive levels (>10^5^ CFU g^−1^ soil), even with continuous no‐till wheat, and *Fusarium* inoculum does not reach an economic threshold [187]. This creates a diagnostic decision rule: no‐till PSF benefit is realizable when soil pH is in the range 5.5–6.8 and SOC > 1.5% [188, 189]. However, in alkaline or carbon‐depleted soils, the residue carryover risk dominates, and no‐till should be paired with mandatory rotation to non‐host crops before its AMF‐network benefit is harvestable [127]. Thus, diversifying plant inputs across time and space is a direct application of indirect PSF principles to break negative feedback cycles and promote positive ones (Figure 4).

### Crop rotation: Disrupting negative feedbacks and building soil fertility

The efficacy of crop rotation depends critically on host range and residue decomposition rates. This classic practice disrupts the buildup of host‐specific pathogens and pests, effectively mitigating negative direct PSF (Table 1 and Figure 4) [190]. Management practices such as crop rotation are potent forces that restructure both the diversity and the underlying assembly processes of rhizosphere bacterial communities, where conventional and organic management generated distinct community patterns [125, 190]. This empirical linkage between farm management and microbiome assembly driving crop diversification as a deliberate PSF tool (Figure 4). Beyond disease suppression, diverse rotations enhance soil carbon and nitrogen stocks [125] and contribute to superior long‐term yield stability and soil health by introducing varied root exudates and litter qualities that support a functionally robust microbial community (Figure 4) [191, 192]. A global meta‐analysis of 122 studies reported that adding one crop to a monoculture rotation increased SOC by 3.6% and total nitrogen by 5.3%. When a legume cover crop (e.g., crimson clover, hairy vetch) was included, SOC gains rose to 8.5% and total nitrogen to 12.8%. Microbial biomass increased by 20.7% and 26.1%, respectively, with effects independent of tillage regime [125]. Thus, diversifying crop across farm‐scale implies direct application of indirect PSF principles to break negative and promote positive feedback cycles (Figure 4). In a 6‐year Wisconsin trial, a 2‐year corn‐soybean rotation supplemented with winter rye cover crop increased particulate organic matter C by 31% and potentially mineralizable N by 27% compared to no‐cover controls [193, 194]. Rotation benefits are maximized when the non‐host period exceeds the pathogen's survival capacity. For *Aphanomyces euteiches* in pea, a 5‐year rotation with non‐host cereals is required; shorter rotations result in disease recurrence within one season [195]. Integrating green manures (e.g., mustard, radish) as short‐season rotation crops can accelerate pathogen suppression through a biofumigation, with incorporation timing critical: tillage immediately after flowering maximizes glucosinolate release [196, 197, 198].

Crop rotation reverses negative PSF by disrupting the amplification cycles of host‐specific pathogens that drive inoculum buildup in monocultures. While conventional rotations primarily attenuate pathogen pressure, the strategic integration of mutualism‐promoting cover crops simultaneously addresses both pathogenic and symbiotic microbial guilds. A representative high‐efficiency sequence utilizes a *Brassica juncea* (mustard) cover crop followed by a 2‐year wheat‐soybean rotation to prime the soil for susceptible cash crops [194, 199]. In this system, mustard biofumigation, applied at 50% bloom and when soil temperatures are >10°C, reduces *Rhizoctonia solani* inoculum by 60%–80% within 14 days via isothiocyanate (ITC) release [198, 200]. This suppression is pH‐dependent; maximal myrosinase activity occurs at pH 5.5–7.0, whereas pH > 7.5 limits glucosinolate hydrolysis and reduces ITC yields by 30%–50% [194, 198]. Subsequent wheat cultivation enriches for *Pseudomonas* carrying *phlD* through benzoxazinoid 2,4‐dihydroxy‐7‐methoxy‐1,4‐benzoxazin‐3‐one (DIMBOA) exudation [201, 202], while the soybean year boosts *nifH* gene abundance 3–5‐fold over continuous wheat and recruits AMF, such as *Rhizophagus irregularis*, via isoflavone and malate exudates [125, 201]. This integrated sequence shifts the rhizosphere pathogen‐to‐mutualist (P:M) ratio from 2.1 in monoculture to 0.4, effectively transitioning the soil from a negative to a positive PSF state.

### Intercropping

Spatial diversification through intercropping introduces simultaneous ecological heterogeneity that disrupts the host‐specific pathogen cycles characteristic of monocultures (Table 1) [203]. This disease‐suppressive effect is highly species‐specific and scales with phylogenetic distance [26, 117]; a meta‐analysis of 108 studies revealed that cereal‐legume intercrops reduce foliar pathogen incidence by 49%, compared to only 21% in cereal‐cereal systems [26]. For example, in wheat‐faba bean intercropping, *Botrytis fabae* (chocolate spot) severity decreases by up to 75% when faba bean density remains below 40% of the total stand. This suppression is driven by the physical interception of spores by wheat stems and the induction of systemic resistance via wheat‐derived volatiles, such as (E)−2‐hexenal [117].

Beyond pathogen dilution, designing systems with phylogenetically distant species selects for complementary microbial functions, such as synchronized nitrogen fixation and phosphorus mobilization, while supporting a greater abundance of natural enemies for pest control [126, 204, 205], while minimizing shared pathogen loads (Figure 4). As a specific application of these agronomic principles, cover cropping offers a direct method to engineer the soil microbiome for enhanced ecosystem functions.

These benefits are primarily mediated through belowground rhizosphere chemical cross‐talk (Figure 4). In maize‐soybean intercropping, soybean roots exude isoflavones (daidzein, genistein) that diffuse into the maize rhizosphere, stimulating AMF hyphal branching and increasing colonization by 22%–35%, even though maize is not the primary isoflavone producer [162, 206, 207]. This reciprocal conditioning represents a synergistic indirect PSF, where the soil environment altered by one species actively enhances the performance and microbial resilience of its heterospecific neighbor (Figure 4).

Practitioners can optimize the rhizosphere residence time of allelochemicals by selecting cover‐crop mulches or compost amendments that increase dissolved organic carbon, thereby outcompeting phenolic acids for soil sorption sites. At the community level, this management approach, exemplified by wheat‐faba bean intercropping, drives a measurable taxonomic shift: ITS2 amplicon sequencing from field trials reveals a significant reduction in *Fusarium* and *Rhizoctonia* operational taxonomic unit (OTU) (from 18.4% to 6.2%) alongside a concomitant increase in the biocontrol agent *Trichoderma* (from 3.1% to 11.8%) [206, 207, 208]. This transition effectively shifts the P:M ratio from 1.8 to 0.7 over two seasons, demonstrating that intercrop design, when calibrated to soil pH and texture, is a tractable tool for steering PSF polarity. Because the PSF experienced by a plant is modified by the density and identity of its neighbors, strategic diversification leverages phylogenetic distance and functional trait dissimilarity to dilute host‐specific pathogens while avoiding the reinforcement of shared negative feedbacks (Figure 4) [44, 119, 209]. Consequently, the design of crop mixtures must prioritize trait divergence to ensure that heterospecific neighbors effectively mitigate the species‐specific inoculum buildup characteristic of conspecific stands.

### Cover cropping

Cover cropping represents a proactive strategy to maintain beneficial PSF during fallow periods. By providing continuous carbon inputs and species‐specific exudates, cover crops foster microbial guilds that reduce soilborne pathogen incidence by an average of 41%, with the Brassicaceae (54% reduction) consistently outperforming the Poaceae and Fabaceae (Table 1) [133, 196, 210]. Leguminous covers (e.g., vetch and clover) additionally provide 70–180 kg N ha^−1^, replacing up to 50% of synthetic nitrogen requirements for subsequent maize while significantly boosting AMF propagule banks [134, 135, 211]. For instance, hairy vetch increases AMF spore density 2.3‐fold, enhancing root colonization in following crops by up to 41%, particularly in phosphorus‐limited soils (Olsen P < 20 mg kg^−1^) [161, 212, 213]. These managed plantings also support larger decomposer populations, such as earthworms, which enhance nutrient cycling and soil aggregation [133, 214].

The suppressive capacity of cover crops like cereal rye is driven by two temporally distinct mechanisms. During active growth, rye roots exude benzoxazinoids (20–50 nmol g^−1^), which directly inhibit fungal pathogens and selectively enrich for *phlD*‐carrying *Pseudomonas* spp. (Figure 4) [187, 198, 200]. This recruitment is highly pH‐sensitive; *phlD* gene copy numbers are 3–5‐fold lower above pH 7.2 [190, 215]. Post‐termination, the decomposing residue provides an additional allelopathic layer of defense through the hydrolysis of DIMBOA [198, 200]. However, these suppressive communities require active maintenance: omitting a rye cover for a single season can reduce *phlD* abundance by 60%, shifting the pathogen‐to‐mutualist (P:M) ratio back toward a negative PSF state (Figure 4) [198, 200]. However, efficacy is tightly governed by soil conditions; myrosinase activity is optimal at pH 6.0–7.0; ITC diffusion requires soil water potential of −0.03 to −0.1 MPa; and glucosinolate content peaks at 50% flowering, making incorporation timing critical [196, 200, 216].

Despite these benefits, the successful implementation of cover crops necessitates managing critical trade‐offs related to termination timing and allelopathy. Late termination maximizes biomass (4–6 t ha^−1^) for weed suppression but risks a 15%–20% yield penalty in rainfed maize due to moisture depletion [217]. Conversely, high rye biomass (>4.5 t ha^−1^) can reduce the emergence of small‐seeded crops by 22% in no‐till systems, a risk mitigated by delaying planting 10–14 days post‐termination [218, 219]. Furthermore, cover crop residues can occasionally serve as a green bridge, providing unintended refuge for pests or pathogens that threaten the primary crop (Figure 4) [217]. Consequently, the transition from fallow‐period engineering to cash‐crop success requires a context‐dependent selection of species and termination methods tailored to local edaphic and climatic constraints.

### Reading the soil: PSF state assessment as a management prerequisite

The efficacy of the agronomic interventions previously discussed is not universal; rather, it is contingent upon the initial biological state of the soil (Figure 4). Conservation tillage, which preserves beneficial fungal networks in a biologically resilient matrix, may inadvertently facilitate pathogen carryover in soils already burdened by high inoculum loads. Similarly, legume‐mediated mutualisms offer diminishing returns in systems where AMF colonization has already reached a saturation plateau. This context‐dependency suggests that the success of an intervention is governed by the biological inertia of the current soil biome, rather than the management history alone (Figure 4). This realization necessitates a “diagnostic‐first” framework, where the soil's feedback polarity is characterized before a management strategy is deployed. Historically, soil management has been reactive; a PSF‐based approach; however, treats the soil as a living system residing in one of three functional states: pathogen‐dominated (negative), biologically impoverished (neutral), or mutualist‐enriched (positive). Deploying an intervention without assessing this state risks wasting metabolic resources or, in worst‐case scenarios, accelerating detrimental feedback loops (Figure 4).

To bridge the gap between theory and the field, we propose a multi‐tiered diagnostic suite using PSF Indicators—quantifiable molecular or functional signatures that predict the direction and magnitude of plant performance. These indicators allow practitioners to “read” the soil across four integrated dimensions: Taxonomic polarity (P:M ratio): Utilizing ITS2 amplicon sequencing, the P:M ratio provides a high‐resolution snapshot of community balance (Figure 4). A P:M ratio > 1 (e.g., dominance of *Fusarium* or *Pythium*) diagnoses a negative PSF state requiring aggressive suppression, while a ratio <0.5 coupled with high AMF read abundance signals a robust positive state [187, 198, 200]. Symbiotic Competency (AMF Colonization): Beyond mere presence, functional occupancy matters [207, 208]. Root colonization levels below 15%–20% in mycorrhiza‐dependent crops identify a mutualism gap where the soil is either depleted or neutral. Conversely, levels exceeding 40%–50% indicate an established positive PSF state where management should shift from restoration to maintenance [120, 177, 178, 179]. Functional Genomic Potential (qPCR Panels): Targeted quantification of key biosynthetic genes, including *nifH* (nitrogen fixation), *phlD* (DAPG‐mediated suppression) [198, 200]. and *phoD* (phosphorus mineralization) [220], reveals the soil's functional toolkit (Figure 4) [190, 215]. A lack of these markers alongside high pathogen DNA loads confirms a critically negative state that necessitates multi‐tiered biological intervention.

Biophysical Capacity (SOC and microbial biomass carbon (MBC): SOC below ~1.5% and MBC below ~150 µg C g^−1^ serve as baseline indicators of biological energy. Regardless of community composition, levels below these thresholds indicate a system lacks the carbon fuel required to sustain positive feedbacks [177, 178, 179]. These four tiers differ in analytical demand: P:M ratio and AMF colonization require sequencing and microscopy, respectively, while SOC and MBC are available through standard commercial soil testing, allowing diagnosis to begin with accessible indicators and be refined as omics‐based data become available. The PSF framework extends existing diagnostic practice by providing a holistic, multi‐indicator view of the entire biotic community balance rather than a single target pathogen. The following sections examine how resource availability and microbial niche construction add further diagnostic dimensions to refine this state assessment.

## 6 RESOURCE AVAILABILITY AS AN ACTIVE LEVER FOR STEERING PSF

The direction and magnitude of PSF are not intrinsic properties of plant genotypes or microbial strains; rather, they are emergent properties of the abiotic resource landscape. Nutrient stoichiometry, hydraulic potential, and light regimes function as rigorous environmental filters that dictate the successional trajectory of microbial guilds (Figure 4). By managing these filters, practitioners can determine whether mutualistic or pathogenic outcomes dominate, effectively deciding if a feedback loop becomes self‐reinforcing or self‐limiting. Critically, these abiotic variables are manageable levers: irrigation scheduling, fertilizer placement, and canopy architecture are deliberate interventions in PSF kinetics (Figure 4). This principle is starkly illustrated by the observation that in soil actinobacteria such as *Micromonospora*, cultivation conditions, not phylogenetic relatedness, are the primary determinant of secondary metabolite output [190, 215]. Hence, agronomic manipulation of the soil physicochemical environment can directly reprogram the metabolome that drives PSF chemistry, independently of taxonomic shifts. A practitioner who recognizes the phosphorus thresholds that suppress arbuscular mycorrhizal symbiosis, or the soil water potential that favors antagonistic *Streptomyces* over oomycete pathogens like *Phytophthora*, possesses a zero‐cost toolkit for state‐steering. This section moves the resource‐PSF relationship from descriptive observation to a prescriptive protocol, outlining how to navigate between PSF states via precision resource management.

### Nutrient management: From selective filter to inoculation enabler

Soil fertility acts as the primary arbiter of whether microbial symbioses deliver a net fitness benefit. According to the trade‐off theory of mycorrhizal evolution, plants allocate carbon to mutualists only when the nutrient return on investment exceeds the metabolic cost [211]. Consequently, a fertilizer regime that maximizes short‐term yield may inadvertently suppress the mutualistic community essential for long‐term stability, creating a negative PSF legacy that only becomes visible across multiple rotations (Figure 4).

Under phosphorus limitation, AMF can supply up to 90% of plant phosphorus (P) demand under limitation (Olsen P < 20 mg kg^−1^ soil) [221], maintaining a strong positive PSF and a P:M ratio typically below 0.5 [44, 213, 222]. However, as soil P rises, the plant's carbon subsidy to the symbiosis declines. Above a critical threshold of 60 mg kg^−1^ Olsen P, AMF colonization often falls below 15%, rendering the symbiosis functionally negligible [207, 208]. This has profound implications for biologicals: applying AMF inoculants into high‐P soils (P > 50 mg kg^−1^) is fundamentally incompatible, not due to product failure, but because the host plant will not sustain the carbon flux required for hyphal establishment [207]. Thus, diagnostic Olsen P testing is a non‐negotiable prerequisite for successful AMF deployment. Conversely, excessive nitrogen availability can attenuate positive PSF in ectomycorrhizal systems by reducing the mutualistic benefit of nutrient monopolization (Figure 4). For instance, excessive nitrogen and phosphorus can inhibit symbiotic mutualists like AMF and rhizobia, which trade nutrients for plant carbon, while simultaneously promoting parasitic and pathogenic organisms [221, 223]. However, increased nutrient availability can also enhance plant vigor and defenses, potentially neutralizing negative PSF driven by pathogens [95, 224].

Strategic fertilizer placement can reconcile agronomic demand with the preservation of microbial networks. Subsurface banding of P fertilizer (5–10 cm lateral offset, 10–15 cm depth) creates a localized spatial gradient (Figure 4). This allows the bulk rooting zone to remain in a low‐P state, maintaining the mutualistic incentive for AMF, while the concentrated band satisfies the crop's total P requirements via non‐mycorrhizal uptake [220, 225]. In maize, this placement‐maintained AMF root length colonization at 32%–45% compared to 12%–18% under broadcast incorporation, with corresponding yield increases of 8%–15% in low‐to‐medium P soils (Olsen P 10–35 mg kg^−1^) [220, 226]. This effect disappears in high‐P soils (Olsen P > 50 mg kg^−1^), confirming that the benefit is contingent on the plant being in a P‐limited state where AMF provides genuine nutrient return.

Similarly, the temporal pulse of nitrogen (N) dictates the success of diazotrophic colonization. Standard urea applications create a mineral N surge that suppresses *nifH* expression and rhizobial nodulation during the critical 0–14 days colonization window (Figure 4) [227]. Transitioning to slow‐release fertilizers, such as polymer‐coated urea or struvite, attenuates this inhibitory pulse [228]. In chickpea, this approach has been shown to increase nodule biomass by 34% by maintaining sufficient N‐limitation during the nodulation phase while satisfying late‐season demand [227, 228]. Struvite also provides slow‐release phosphorus at pH < 7.0, making it compatible with AMF colonization in acidic soils where soluble P equivalent would suppress symbiosis [227, 228].

The PSF outcome of strategic nutrient management can be summarized through two metrics. First, maintaining Olsen P < 20–35 mg kg^−1^ in the rhizosphere zone (via banded placement rather than broadcast) consistently maintains AMF colonization above 30% and P:M ratios below 0.6, creating and sustaining positive PSF in the mutualist‐functional dimension. Second, using slow‐release or split nitrogen application to keep mineral N below the rhizobial and plant growth‐promoting bacteria (PGPB) suppression threshold (60–80 kg N ha^−1^ at the colonization) preserves the nitrogen‐fixing and disease‐suppressive capacity of the introduced consortium through the first 14–21 days when rhizosphere establishment is irreversibly determined [227, 228, 229]. Nutrient management that fails these two criteria will predictably suppress introduced inoculants regardless of product quality, and this mechanism explains the high field‐trial variability in inoculant performance that has slowed commercial adoption (Figure 4).

### Water management: Irrigation as a microbiome engineering tool

Water availability modulates PSF through three primary mechanisms: (i) diffusional constraints on microbial motility and substrate access; (ii) oxygen availability that selects for aerobic versus facultative anaerobic taxa; and (iii) plant carbon allocation under drought stress, which often increases root exudation of osmolytes and defense compounds (Figure 4) [230, 231]. Strategic irrigation regimes can therefore be deployed to engineer specific rhizosphere assemblages. For instance, deficit irrigation (60% evapotranspiration) has been shown to enrich the rhizosphere for *Streptomyces* spp. carrying siderophore and ACC‐deaminase genes, correlating with a 28% reduction in *Fusarium* wilt [112, 232]. This is driven by a drought‐induced increase in citrate and malate exudation, which selectively stimulates antagonistic actinobacteria while inhibiting pathogenic fungi. This is driven by a drought‐induced increase in citrate and malate exudation, which selectively stimulates antagonistic actinobacteria while inhibiting pathogenic fungi [112, 232]. Conversely, high‐moisture regimes, such as furrow irrigation on heavy soils, foster hypoxic conditions that favor denitrifiers and oomycete pathogens like *Pythium* and *Phytophthora*. Transitioning from surface to drip irrigation reduces hydraulic fluctuations, which in avocado systems has been shown to decrease *Phytophthora cinnamomi* propagules by 70% while simultaneously increasing AMF colonization from 18% to 41% (Figure 4) [230].

The interaction between irrigation regime and microbial inoculation is multiplicative. Drought‐tolerant strains (e.g., *P. putida*, *B. amyloliquefaciens*) exhibit superior rhizosphere persistence under deficit irrigation because the reduced water potential restricts fast‐growing copiotrophic competitors (Figure 4) [233]. Subsequently, deficit irrigation “pre‐selects” a niche that the inoculant is functionally designed to fill, rather than forcing it to compete in the saturated, copiotroph‐rich environment of a full‐irrigation regime [234]. To capitalize on this synergy, the kinetic timing of application is critical [234]. Selective inoculation of inoculants should be applied at the start of deficit irrigation to ensure they enter a selective environment where they have a competitive advantage. Avoiding copiotrophic surges is crucial because applying inoculants 24–48 h before or during full irrigation places the product in a high‐resource environment dominated by native competitors, which significantly lowers establishment rates (Figure 4). Thus, for practitioners, irrigation management is important, but light and canopy decisions also influence PSF dynamics, helping to reduce disease and stress while enhancing mutualist functions.

### Light and canopy architecture: Carbon delivery as a PSF lever

Light availability is the ultimate constraint on PSF, as it dictates the total photosynthate pool available for subterranean allocation. Because maintaining mutualistic networks like AMF or rhizobial nodules requires significant metabolic effort, shading functions as a metabolic shut‐off valve. When light is limited, plants prioritize internal survival over external symbiosis [235, 236], leading to the regression of beneficial microbial guilds regardless of soil inoculant presence [208]. The interaction between light and PSF is mediated by a species' ecological strategy. Shade‐tolerant species have evolved high carbon‐use efficiency, allowing them to maintain positive (or less negative) PSF even under closed canopies by preserving microbial associations with minimal carbon expenditure [237]. In contrast, light‐demanding pioneer species utilize high‐irradiance environments to flood the rhizosphere with carbon. This rapid niche construction builds a supportive microbial community that further accelerates growth, creating a self‐reinforcing dominance in forest gaps or open fields [237].

Canopy architecture, defined by leaf area index (LAI), vertical foliage distribution, and leaf angles, is the primary determinant of light interception efficiency and, by extension, the rhizosphere carbon budget (Figure 4) [208, 236, 237, 238]. Management interventions that optimize light penetration can selectively enhance mutualist recruitment. In strawberry systems, plastic mulches that reflect photosynthetically active radiation (PAR) into the lower canopy have been shown to increase AMF colonization and total yield [239]. In tomato, pruning to a dual‐stem architecture increases the root‐to‐shoot ratio, elevating rhizosphere microbial biomass carbon and reducing the severity of *Xanthomonas* spp. infections [184], demonstrating that canopy pruning is simultaneously a PSF intervention and a disease management tool, operating through the shared mechanism of root carbon delivery (Figure 4) [240]. In cereal crops like wheat, upright leaf architectures improve light penetration to the base of the plant. This increases the pool of non‐structural carbohydrates available for exudation, sustaining more diverse and disease‐suppressive soil communities even at high planting densities [241].

However, the interplay between canopy‐mediated light gradients and carbon delivery is particularly evident during forest succession and in managed agroecosystems. In early‐successional tropical forests, gap‐induced increases in light availability prompt seedlings to shift biomass allocation toward roots, enhancing nutrient foraging capacity (Figure 4) [237, 240]. Early‐ and mid‐successional species display greater plasticity in root‐to‐shoot ratios under elevated light, facilitating rapid exploitation of transient resource pulses [237, 240]. These allocation shifts amplify carbon inputs to soil, accelerating microbial turnover and nutrient release that reinforce PSF‐driven community assembly.

Light and canopy architecture are the functional controls of a powerful ecological engine (Figure 4). By determining the “metabolic currency” available for rhizosphere delivery, canopy structure regulates the feedback polarity that leads to either species dominance or stable coexistence. As we move toward an integrated understanding of plant‐soil‐atmosphere interactions, the role of canopy‐driven carbon allocation will remain central in predicting the resilience and composition of future ecosystems.

### Integrated resource management as a PSF state steering protocol

The three resource levers, nutrient stoichiometry, hydraulic potential, and photonic flux, do not operate in isolation; rather, they converge synergistically upon a single biological substrate: the carbon economy of the plant–microbe interface (Figure 4). Plants actively tailor their root exudates profiles in response to specific resource deficits, such as secreting targeted organic acids under phosphorus or iron limitation to mobilize nutrients and recruit suppressive microbial taxa [242]. Building SOC through strategic amendments further buffers this system, expanding the functional elasticity of the rhizosphere by improving water retention and stabilizing nutrient supply (Figure 4) [35].

Consequently, the optimization of PSF follows a biological analog to Liebig's Law of the Minimum [243]. A crop subjected to strategic nutrient limitation, yet maintained at optimal hydraulic and photonic thresholds, operates at peak mutualistic investment, as the aligned environment makes carbon allocation economically rational. Conversely, the mismanagement of any single abiotic axis, such as severe shading or hypoxic irrigation, can collapse the root carbon economy, overriding the mutualist‐promoting effects of the other levers [244].

This multi‐axis dependency necessitates a diagnostic, systems‐level approach to PSF management. By positioning agronomic practices not as passive background conditions, but as active, tunable levers, practitioners can establish the requisite abiotic foundation for biological interventions (Figure 4). In the following section, we examine emerging strategies, microbiome‐assisted selection, rewilding, and vertical transmission, that aim to restore and amplify the plant's intrinsic capacity to promote positive PSF.

## 7 INTEGRATING PSF TOOLBOX VIA COMPLEX INTERPLAY OF MICROBIOME‐ASSISTED BREEDING, HOLOBIONT, AND REWILDING STRATEGIES

While agronomic practices shape the edaphic habitat and resource availability tunes the environmental filters, the plant genotype remains the central biological actuator of PSF. Historically optimized for yield under high‐input regimes, modern crop breeding has inadvertently selected against the metabolic traits required to sustain beneficial microbial partnerships [35, 83, 245]. Plant domestication and intensive agriculture have reduced plant genetic diversity and disrupted coevolved plant‐microbiome alliances, a “domestication syndrome” that homogenizes and functionally depletes crop microbiomes (Figure 5A) [79, 246]. Consequently, modern elite lines frequently exhibit a “domestication discount,” characterized by attenuated AMF responsiveness and a diminished genetic capacity to actively recruit disease‐suppressive taxa [33, 79, 247]. To reclaim these lost symbiotic functions, the holobiont concept, which conceptualizes the host plant and its associated microbiome as a singular, co‐evolving ecological unit, offers a transformative breeding paradigm [245, 248, 249].

**Figure 5:**
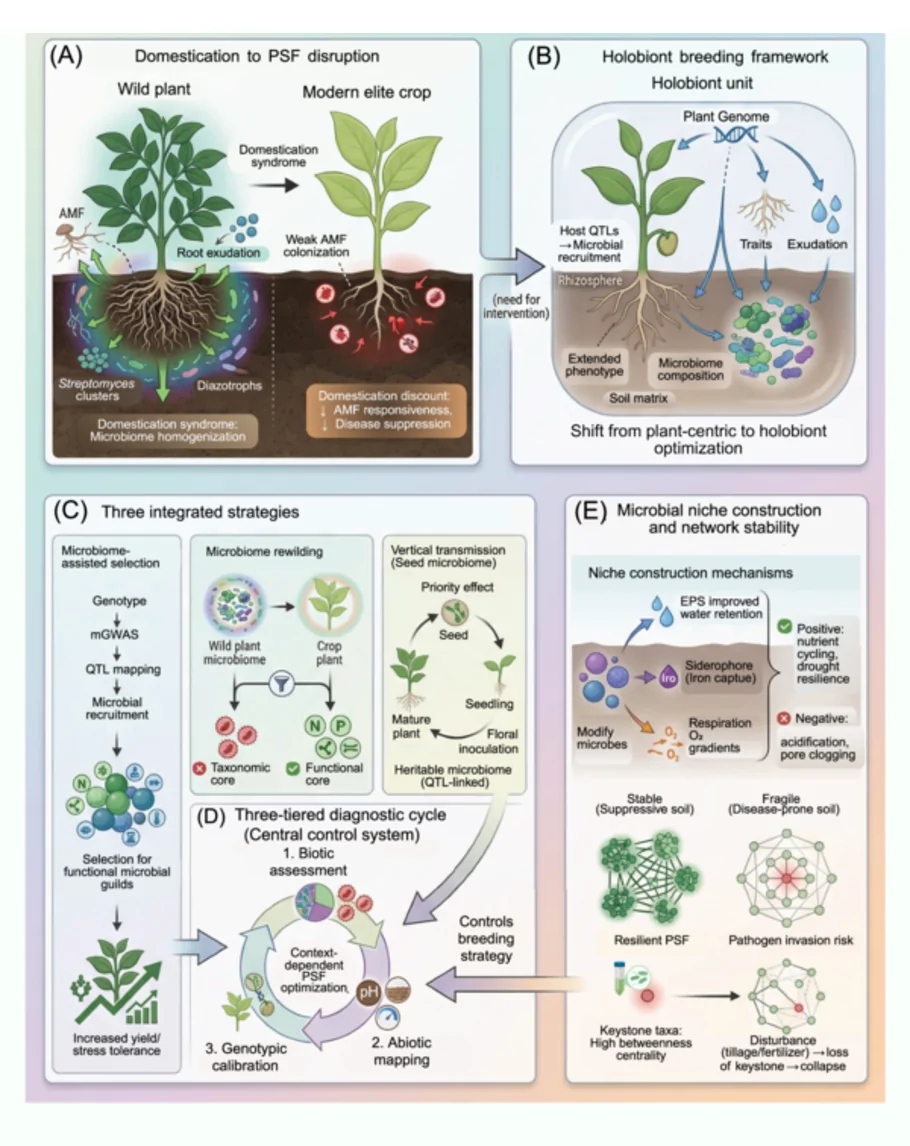
Engineering positive plant‐soil feedback (PSF) through holobiont breeding, microbiome rewilding, and microbial niche construction. (A) Domestication Syndrome‐based PSF Disruption: Illustrates the “domestication syndrome” where wild plants with robust root exudation and diverse microbial associations (e.g., arbuscular mycorrhizal fungal (AMF), *Streptomyces* clusters, Diazotrophs) are bred into modern elite crops. This process often results in a “domestication discount,” characterized by weakened AMF colonization, microbiome homogenization, and decreased responsiveness to beneficial microbes and disease suppression. (B) Holobiont Breeding Framework: Proposes a paradigm shift from plant‐centric breeding to optimizing the entire “holobiont unit.” It highlights the need for intervention by mapping how the plant genome (Host QTLs) controls root traits and exudates. These plant‐driven factors act as an extended phenotype, directly influencing microbial recruitment and microbiome composition in the rhizosphere and surrounding soil matrix. (C) Three integrated strategies: Actionable pathways for microbiome optimization; microbiome‐assisted selection; Utilizing genotype data, mGWAS, and QTL mapping to select for functional microbial guilds, ultimately driving increased yield and stress tolerance. Microbiome rewilding: the targeted transfer of the wild plant microbiome to crop plants. It emphasizes establishing a functional core (promoting N and P cycling) rather than just a taxonomic core. Vertical transmission (Seed microbiome): Harnessing priority effects by establishing a heritable, QTL‐linked microbiome. This is achieved through pathways like floral inoculation of the mature plant to pass beneficial microbes directly to the seed and subsequent seedling. (D) Three‐Tiered Diagnostic Cycle (Central Control System): A continuous feedback loop that controls the breeding strategy. It relies on (1) Biotic assessment, (2) Abiotic mapping (e.g., pH, moisture), and (3) Genotypic calibration to achieve context‐dependent PSF optimization. (E) Microbial niche construction and network stability: Demonstrates how microbes modify their environment (e.g., via EPS for water retention, siderophores for iron capture, or respiration altering O_2_ gradients). These modifications can have positive (drought resilience) or negative (pore clogging) effects. The panel further contrasts network stability: Stable (Suppressive Soil) as a highly interconnected, resilient network that promotes positive PSF. Fragile (Disease‐prone soil) indicates a vulnerable network at risk of pathogen invasion. It highlights the critical role of keystone taxa” (nodes with high betweenness centrality) and how agricultural disturbances (tillage/fertilizers) can lead to the loss of these keystones and subsequent ecosystem collapse. EPS, extracellular polymeric substances; mGWAS, microbiome genome‐wide association studies; QTL, quantitative trait loci; N, nitrogen; P, phosphorus.

This framework shifts the agronomic goal from optimizing the plant in isolation to maximizing the fitness and function of the integrated plant‐microbe partnership (Figure 5A). By identifying and introgressing the genetic loci that govern microbial recruitment, breeders can develop cultivars explicitly calibrated to engineer their own soil health. To operationalize this holobiont framework, three complementary strategies are currently emerging to restore the plant's intrinsic capacity to drive positive PSF.

### Microbiome‐assisted selection

Microbiome‐assisted selection transcends traditional agronomic breeding by treating microbial recruitment not as an environmental variable, but as a heritable, extended plant phenotype (Figure 5A). Rather than phenotyping solely for aboveground yield or standability, breeding lines are evaluated for their rhizosphere competency, and their intrinsic genetic capacity to assemble specific, functional microbial guilds linked to nitrogen fixation, drought tolerance, or pathogen suppression [250, 251]. A promising framework for this effort is the holobiont concept, which views the plant and its microbiome as a cohesive evolutionary unit (Figure 5B,C) [83, 252]. This holobiont‐centric breeding is operationalized through microbiome GWAS. This high‐throughput statistical genomic approach allows breeders to map specific host quantitative trait loci (QTLs) directly to the abundance and activity of target microbial taxa (Figure 5C) [81, 247]. By identifying the genetic architecture that governs these interactions, breeders can explicitly select for symbiotic traits. For example, in maize, the targeted selection of a *TPX2* domain‐encoding gene has been shown to modulate lateral root architecture, which specifically enriches for growth‐promoting *Massilia* species under nitrogen‐limiting conditions [137]. Similarly, in tomato, the introgression of a specific transcription factor actively alters the root exudomic profile to preferentially recruit *Streptomyces* populations [253]. Experiments show that artificial selection on rhizosphere microbiomes can increase seed production under severe salt stress by ~55%–200% and alter ion homeostasis (e.g., halving leaf Na^+^) in cereals models [254, 255]. Successive passaging can yield robust, invasion‐resistant leaf microbiomes adapted to specific host genotypes and habitats [256]. However, theory and models highlight strong constraints: effective holobiont‐level selection requires high microbiome heritability, relatively fast host generation times, and control over horizontal microbial acquisition [257]. These studies show that breeding microbiomes can rapidly and substantially improve plant stress tolerance and performance, especially under controlled transmission (Figure 5). Yet stability across environments, transmission fidelity, and managing complex eco‐evolutionary dynamics remain key challenges before routine deployment in crops to achieve positive PSF.

### Microbiome rewilding

Microbiome rewilding seeks to restore adaptive plant‐microbe partnerships lost during domestication by reintroducing microbial consortia from wild crop progenitors [246, 257, 258]. However, this strategy requires caution, as wild plants, often adapted to pathogen‐rich, early‐successional environments, can harbor core microbiomes that include soil‐borne pathogens [21, 29]. While the concept of a core microbiome, taxa consistently detected across multiple host accessions, is a popular target for rewilding, its application is often hindered by significant definitional baggage. Operational definitions based on prevalence thresholds (e.g., taxa present in 80%–100% of samples) are inherently arbitrary and sensitive to sequencing depth, rarefaction strategies, and geographic sampling breadth [259, 260, 261]. Furthermore, wild progenitors adapted to pathogen‐rich, early‐successional environments often harbor “core” taxa that include latent soilborne pathogens [45, 262, 263]. Consequently, a taxonomic core defined by conserved OTU is an inadequate target for PSF‐based rewilding (Figure 5C). Instead, we propose a shift toward identifying a functional core: a conserved suite of metabolic capabilities, such as nitrogen fixation, phosphate solubilization, and antibiosis, that are consistently present across progenitor microbiomes regardless of taxonomic composition [259, 260, 261]. Selecting for functional capacity rather than taxonomic identity sidesteps the pathogen problem, ensuring that rewilding programs retain beneficial guilds while excluding prevalent but deleterious taxa [252].

By exploring these relationships, we can breed crops that cultivate strong microbial partnerships, essential for resilient and sustainable agricultural systems.

### Vertical transmission

The seed microbiome represents a conserved, vertical transmission channel that provides the developing seedling with its initial inoculum. This heritable microbial suite offers a unique opportunity to prime the next generation for positive PSF from the moment of germination (Figure 5C) [248, 251]. Evidence shows that seed‐borne microbes can influence seedling vigor, nutrient uptake, and stress resistance [189]. Unlike soil‐borne recruitment, which is subject to intense competition and environmental filtering, seed‐borne endophytes benefit from an ecological priority effect [47, 264]; because they are present at the onset of sprouting, they can rapidly colonize the emerging tissues and establish a dominant, protective niche before soil‐borne pathogens can intervene (Figure 5C) [90, 265].

Recent breakthroughs in *in‐planta* engineering demonstrate that the seed microbiome is a tunable target. Floral inoculation, the introduction of beneficial bacteria during the flowering stage, allows for the deliberate “loading” of the next generation's microbiome. For instance, in wheat, inoculation with *Paenibacillus polymyxa* successfully achieved vertical transmission, resulting in a 15% increase in seedling emergence and a 9% boost in final grain yield under field conditions [264, 266]. In pepper, using floral sprays to introduce *Paraburkholderia phytofirmans* (PsJN) proved that complex endophytes can be reliably vectored through the seed to enhance progeny vigor [267]. Similarly, seed‐applied consortia of *Enterobacter*, *Pantoea*, *Pseudomonas*, and *Stenotrophomonas* increased nitrogen uptake and yield by 11% across diverse site‐years, confirming the stability of these engineered legacies [264, 268].

The composition of these seed‐borne communities is not random; it is a heritable trait of the host plant. Certain studies focused on seed‐associated microorganisms (endophytes) that can be passed down from one generation to the next [90, 269]. In maize inbred lines, the structure of the seed bacterial community exhibits significant heritability (*H* ^2^ = 0.18–0.34), with specific QTL mapped to the abundance of beneficial taxa such as *Sphingomonas* and *Methylobacterium* [269]. This genetic foundation suggests that breeding programs can actively select for high‐transmission genotypes that consistently pass down a protective microbial shield.

By integrating the vertical transmission of functional endophytes with microbiome‐assisted selection and rewilding [268], we move toward a breeding pillar where crops are no longer passive recipients of their soil environment. Instead, cultivars are developed as active architects of their own rhizosphere. Through the deliberate management of host genetics and transgenerational microbial legacies, we can ensure that future crops are born into a supportive ecological context, fundamentally shifting PSF outcomes toward long‐term resilience and productivity (Figure 5C,D).

### Navigating context‐dependency: The three‐tiered diagnostic cycle

The central challenge in implementing PSF management is not the existence of context‐dependency, but the prevailing tendency to treat it as an anomaly rather than the fundamental rule [221]. Success is rarely determined by the intrinsic quality of an intervention; it is determined by the precision with which that intervention is calibrated to local edaphic and socio‐economic realities [270]. Soil legacies do not act in isolation; their effects are filtered through soil texture, mineralogy, and climate. Consequently, the era of universal agronomic prescriptions is over, replaced by adaptive, diagnostic frameworks that treat every field as a unique bio‐physical entity (Figure 5C,D) [262, 271, 272].

We propose a fundamental operational shift: the selection of the plant partner must follow, not precede, the diagnosis of the soil. The plant genotype is the ultimate biological lever, but it remains inert, or becomes counterproductive, if activated in ignorance of the soil's state. A cultivar bred to recruit *Streptomyces* for suppression will fail in anaerobic or high‐pH environments where actinobacteria cannot establish [112, 232, 253]. Similarly, selecting for nitrogen‐fixing partnerships is economically futile if (e.g., *nifH* [125, 201]) qPCR profiling confirms that diazotrophic capacity is not the limiting factor (Figure 5C,D).

To resolve this, PSF management must be structured as a three‐tiered diagnostic cycle:

- Biotic state assessment: Characterize the soil's microbial memory, identifying existing functional guilds and pathogen inoculum loads.
- Abiotic environmental mapping: Define the physical and chemical stage—texture, pH, and hydraulic potential—that will act as the filter for microbial survival.
- Genotypic calibration: Deploy the specific genetic strategy (cultivar or holobiont breeding target) engineered to recruit and reinforce the desired community within the identified biotic and abiotic constraints.

Transitioning from ecologically optimal to economically viable requires a radical integration of ecology, economics, and policy. Adoption hinges on profitability at the spatio‐temporal scales relevant to the producer (Figure 5C,D) [273, 274, 275]. Complex interventions, such as high‐diversity intercropping, often clash with legacy administrative and technological regimes [276, 277]. Therefore, PSF policy must innovate alongside the science, utilizing incentive structures and rigorous cost‐benefit quantifications to ensure that sustainable soil engineering is also the most profitable path [278, 279].

Operationalizing this three‐tier framework necessitates a new class of research tools that bridge scales and disciplines [158, 280]. To move beyond correlative observations, we must adopt state‐of‐the‐art causal inference methods, such as structural equation modeling, to disentangle the multiple pathways linking environmental gradients to crop outcomes [281, 282, 283]. Spatially explicit process‐based models, integrated with dynamic cloud‐based databases and on‐farm data streams, can generate locally relevant management responses rather than generic recommendations [263, 284, 285, 286, 287]. Crucially, the development and validation of these tools require a multi‐stakeholder approach. Living labs, where farmers, ecologists, and industry partners collaborate, accelerate the co‐production of knowledge and shorten the innovation‐to‐adoption timeline [288, 289, 290, 291].

This vision reframes the plant genotype not as a passive recipient of services, but as the final, calibrated output of a diagnostic process [280, 292]. By integrating biotic, abiotic, and genetic tiers into a unified adaptive management cycle (detailed in the section “from ecological insight to PSF predictive principle: a proposed model”), we transform PSF management from a game of probabilistic outcomes into a strategy of targeted intervention. This transition sets the critical stage for our next inquiry: if the plant is the lever, how do the microbes themselves, as active habitat engineers, stabilize or subvert the very feedback loops we seek to control?

## 8 MICROBIAL NICHE CONSTRUCTION AND MODIFICATION FEEDBACKS IN PSF

Microorganisms are not merely passive inhabitants of the soil matrix; they are their primary architects. Through metabolic fluxes, extracellular secretions, and communal interactions, microbes continuously reorganize the physicochemical landscape, altering pH, aggregate stability, and nutrient diffusion. These modifications generate autonomous feedback loops that intersect with, and occasionally subvert, plant‐driven PSF [293, 294, 295]. The potency of this engineering is evident in laboratory evolution: populations of *Pseudomonas fluorescens* returned to their ancestral environment after only 100 generations exhibit significant fitness declines, proving they had adapted to a niche they themselves had constructed (Figure 5E) [296, 297].

A primary mechanism of positive niche construction is the secretion of Extracellular Polymeric Substances (EPS). Under desiccation stress, *Pseudomonas* spp. upregulates EPS production to preserve aqueous‐phase connectivity, extending their activity window and maintaining nutrient diffusion even at low matric potentials [298, 299, 300, 301, 302]. This EPS‐mediated habitat modification directly benefits the producing organisms by extending their activity window during dry periods. However, microorganisms can also alter their local environment in ways that reduce their overall fitness (Figure 5E) [303]. In a laboratory microcosm experiment with a high‐glucose environments, soil bacteria can acidify their local medium to pH 3.5 inducing their own extinction, a stark reminder that traits conferring a short‐term competitive advantage can become maladaptive when environmental thresholds are breached [36, 304]. While EPS provides a hydraulic buffer, its progressive accumulation can clog soil pores and inhibit gas exchange, eventually suppressing the very activity it was meant to protect (Figure 5E) [305]. The prevalence of such direct negative feedback in complex natural soil communities remains uncertain, but their existence underscores that microbial engineering of soil habitat is not inherently beneficial. Its outcome depends on the magnitude, duration, and context of modification.

In diverse communities, one organism's niche construction is another's selective pressure [297]. This is most visible within soil aggregates, where microbial respiration creates steep oxygen gradients [306, 307]. These anoxic interiors select for facultative anaerobes, driving a redox‐driven succession that favors denitrifiers, potentially leading to significant nitrogen loss in agroecosystems [307, 308]. The same aggregate structure that protects organic matter from decomposition in one microsite simultaneously creates anoxic refugia for entirely different metabolic guilds [308]. Furthermore, fungi are generally considered more drought‐tolerant than bacteria due to their hyphal architecture and osmotic adjustment mechanisms [307, 308, 309]. Some fungi further modify their environment by producing hydrophobic compounds (hydrophobins, mycospocrins) that reduce water infiltration and create drier microsites [310, 311]. This hydrophobic niche construction may provide a competitive advantage over bacteria during dry periods, but could also suppress bacterial‐mediated nutrient cycling when water becomes limiting, a trade‐off with implications for PSF in rainfed agriculture (Figure 5E) [311].

Furthermore, the production of siderophores (high‐affinity iron chelators) introduces a complex social dilemma. While secretion solubilizes ferric iron for the producer, it creates a public good vulnerable to exploitation [312]. Cheater strains, which possess receptors but lack the biosynthetic machinery for siderophores, can outcompete producers, destabilize the cooperative trait and alter the functional stability of the soil microbiome [313, 314, 315, 316, 317, 318]. The balance between producers and cheaters depends on spatial structure, diffusion rates, and iron availability, all modifiable by agronomic practices.

Niche construction theory shifts our perspective from adding inputs to managing systems. Implementing PSF‐driven agriculture requires an integrated, context‐aware framework that acknowledges these ecological trade‐offs. The goal is to move beyond the search for a single microbial product and instead embrace the principles of habitat engineering (Figure 5E) [319, 320, 321], and developing DSFs that tailor recommendations to local soil conditions and farming contexts [322]. By aligning human management with the natural architectural tendencies of microbes, we can design cropping systems that generate self‐reinforcing cycles of soil health. This transforms PSF knowledge from a theoretical blueprint into a set of actionable ecological principles, bridging the gap between fundamental microbial ecology and sustainable, resilient agronomy.

### Co‐occurrence networks, keystone taxa, and the ecological basis of PSF stability

The interaction dynamics within the soil microbiome, encompassing public goods dilemmas, redox‐driven guild partitioning, and indirect feedbacks, do not operate between isolated taxon pairs. Instead, they are embedded within highly complex, community‐level networks. Ultimately, it is the topology of these networks that dictates whether a PSF state is structurally suppressive and resilient, or fragile and susceptible to pathogen invasion. Network ecology provides the analytical framework to map these topologies and decode the emergent properties that sustain soil health (Figure 5E).

The architecture of resilience is based on comparing modularity and nestedness. Co‐occurrence network analysis (utilizing compositional pipelines such as SparCC or SPIEC‐EASI) translates amplicon sequencing data into interaction graphs, where nodes represent taxa and edges represent statistically non‐random associations [261, 321, 323]. The macroscopic structural properties of these graphs carry profound biological meaning. In addition, modularity act as a buffer and reflects the degree to which a network is organized into densely connected, semi‐independent sub‐clusters (*e.g*., nitrogen fixers, phosphate solubilizers, and antibiosis producers) [261]. Suppressive soils consistently exhibit highly modular networks [190, 261]. This compartmentalized architecture buffers the broader community against localized perturbations, preventing the failure of a single guild from cascading through the system [155, 324]. Meanwhile, nestedness reflects the vulnerability. Conversely, networks characterized by high nestedness, where specialist taxa preferentially link only to generalist hubs, are architecturally fragile. The loss of a single hub taxon can trigger disproportionate community collapse. Rooted in the May‐Wigner theoretical model, empirical soil networks maintain stability not through hyper‐connectivity, but through this non‐random, modular segregation of duties (Figure 5E) [325].

Comparative co‐occurrence network studies across PSF states consistently show that suppressive soils harbor more modular, less nestedness‐dependent networks than disease‐conducive soils [155, 324], providing a network‐level explanation for why suppressive states resist pathogen invasion: the modular guild structure creates functional redundancy that prevents single‐node failure from propagating across the network (Figure 5E) [325].

Within these networks, certain nodes exert an influence on community structure that is vastly disproportionate to their numerical abundance [165]. These are keystone taxa [155]. Computationally identified by their high “betweenness centrality” combined with low relative abundance, keystone taxa act as the critical bottlenecks controlling the flow of information and metabolic resources [155]. In agricultural soils, these hubs are frequently low‐abundance actinobacteria or proteobacteria that regulate multiple associated taxa by secreting broad‐spectrum antibiotics, siderophores, or volatile organic compounds [326, 327]. Their relevance to management is direct: intense agronomic disturbances (e.g., fumigation, deep tillage, high‐dose synthetic nitrogen) can easily fracture these keystone hubs. When a hub is lost, the network shifts from a stable, modular state to a fragile, nested state prone to pathogen invasion, even if the overall microbial diversity appears unchanged (Figure 5E).

These network dynamics converge on a single, management‐defining principle: PSF stability is an emergent property of network topology, not of individual taxonomic presence. A soil community can host all the prerequisite biocontrol agents, *phlD*‐expressing *Pseudomonas* [190], AMF [175], *Streptomyces* [253], and *Trichoderma* [206, 207, 208], yet completely fail to suppress disease if the interaction network is dominated by competitive exclusion rather than modular cooperation. Taxa operating in isolation cannot produce the emergent, system‐level functions (pH buffering, iron sequestration cascades) that require coordinated network activity (Figure 5E). Consequently, next‐generation diagnostics must measure network topology (via platforms like QIIME2 integrated with NetCoMi), and next‐generation inoculants must be designed to restore guild structure and hub‐taxon function, rather than merely introducing isolated strains [306]. This predicts that keystone taxa abundance and network centrality, not total microbial diversity, are the diagnostic indicators most predictive of PSF state, a hypothesis now testable with routine amplicon sequencing combined with network analysis pipelines (QIIME2 + CoNet or NetCoMi) [306].

The preceding sections have established three diagnostic tiers, biotic state, abiotic environment, and plant genotype, that collectively define a soil's initial PSF baseline. Current section has now introduced the fourth, most dynamic tier: Microbial Niche Construction and Network Topology. Because soil communities are self‐modifying, the PSF state is never static; it is continually rewritten by its microscopic architects. What remains missing from the current literature is the integration of these four tiers into a singular, operational logic. The practitioner requires a framework that dictates not only how to diagnose each dimension, but how to select, sequence, and dynamically adapt interventions based on that integrated diagnosis. Synthesizing these dimensions into a cohesive, predictive management cycle is the focus of the next section.

## 9 FROM ECOLOGICAL INSIGHT TO PSF PREDICTIVE PRINCIPLE: A PROPOSED MODEL

To build a scientifically rigorous agricultural management model, we must first demonstrate that PSF are not merely descriptive ecological concepts, but predictable and quantifiable forces. Recent evidence underscores that this legacy is complex, and a unique balance of pathogens and mutualists is the foundation of species‐specific feedback strengths that can range from strongly negative to robustly positive evidence by multiple foundational studies [20, 44, 214, 223, 328, 329, 330, 331]. In a meticulous 7‐year field experiment across multiple crop species, researchers confirmed that crops condition their soil with unique, species‐specific balances of pathogens and mutualists (Figure 6) [332, 333]. For instance, soybean (*Glycine max*) frequently drives strong negative feedback by accumulating host‐specific fungal pathogens, whereas lentils foster robust positive feedbacks characterized by a strong negative correlation between plant biomass and pathogen load [333, 334]. Crucially, null models that ignore PSF effects fail dramatically to predict plant community outcomes [318]. This evidence forces a paradigm shift: agricultural management can no longer focus on the plant in isolation; it must manage the entire plant‐microbe‐soil nexus.

**Figure 6:**
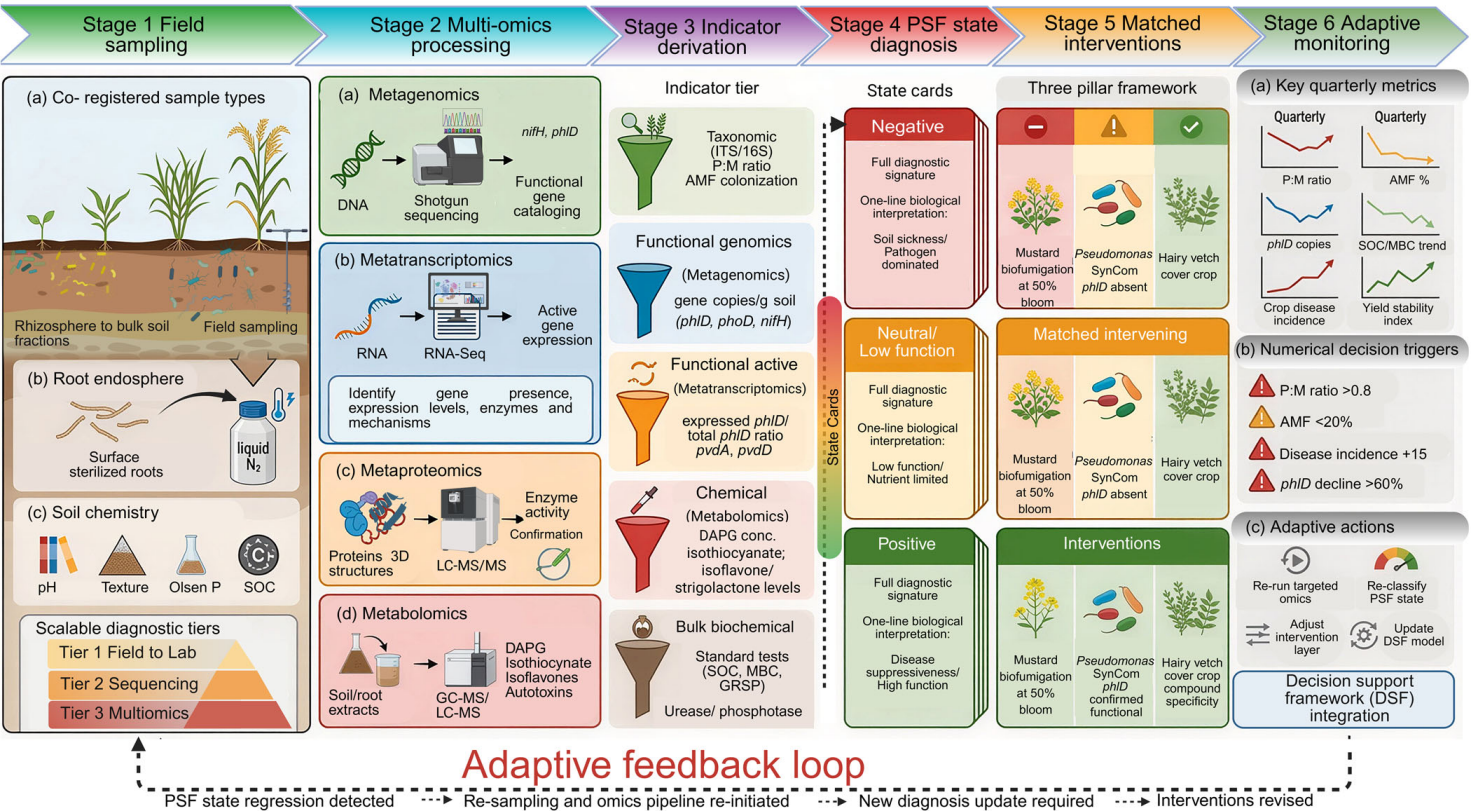
A meta‐omics‐to‐management pipeline for PSF‐informed agricultural decision‐making. The pipeline operationalizes the diagnostic‐intervention‐adaptation cycle across six sequential stages. Stage 1 (Field sampling) illustrates co‐registered collection of rhizosphere soil, root endosphere tissue, and soil physicochemical data at three crop developmental stages (seedling, tillering, grain‐fill), with a scalable diagnostic entry‐point framework spanning farm‐laboratory tests (Tier 1) through full meta‐omics (Tier 3). Stage 2 (Multi‐omics processing) depicts four analytically non‐redundant omics layers, metagenomics (community functional gene catalogue), metatranscriptomics (active gene expression), metaproteomics (enzymatic output confirmation), and metabolomics (net soil chemical fingerprint), emphasizing that gene presence, expression, enzymatic activity, and chemical outcome are non‐interchangeable information layers. Stage 3 (Indicator derivation) translates each omics layer into five quantitative indicator tiers: taxonomic (pathogen‐to‐mutualist ratio, AMF colonization %), functional‐genomic (*nifH*, *phlD*, *phoD*, *amoA* copy numbers g^−1^ soil), functional‐active (expressed *phlD*: total *phlD* ratio; *pvdA*/*pvdD* siderophore expression), chemical (DAPG concentration; isothiocyanate profile; isoflavone/strigolactone levels), and bulk biochemical (SOC, MBC, GRSP, enzyme activities). Stage 4 (PSF state diagnosis) classifies soils into three feedback states, Negative PSF (P:M > 1.0; pathogen‐dominated), Neutral/Low‐Function PSF (P:M 0.5–1.0; biologically depleted), and Positive PSF (P:M < 0.5; mutualist‐enriched), based on combined indicator thresholds. Stage 5 (Matched intervention) maps each diagnosed state to a state‐specific Three‐pillar intervention protocol specifying actions across Plant Partner engineering (cultivar selection, exudate modification), Soil habitat management (biofumigation, rotation, tillage), and biological agent deployment (SynCom inoculants, AMF consortia, PGPB). Stage 6 (Adaptive monitoring) defines quarterly key metrics, numerical decision triggers for re‐intervention (e.g., P:M ratio rising above 0.8; *phlD* decline > 60%), and adaptive actions feeding into the DSF. A dashed feedback arrow from Stage 6 to Stage 1 denotes that PSF state regression detected in monitoring re‐initiates the full sampling and omics pipeline, creating a closed adaptive learning cycle. The decision threshold table (bottom panel) operationalizes state classification across five indicators for direct practitioner reference. DSF, Decision‐Support Framework; DSF, Decision‐Support Framework; DAPG, 2,4‐diacetylphloroglucinol; GRSP, glomalin‐related soil protein; MBC, microbial biomass carbon; P:M, pathogen‐to‐mutualist ratio; PSF, plant‐soil feedback; PGPB, plant growth‐promoting bacteria; SOC, soil organic carbon; SynCom, Synthetic microbial communities.

### From prediction to proactive management: A three‐pillar framework

With the predictability of PSF established, we can transition from reactive treatments to proactive steering. Therefore, effective management involves strategically amplifying positive signals (like beneficial microbes and nutrient cycling) while suppressing negative ones (like pathogen accumulation) (Figure 6). The core objective is to strategically amplify positive biological signals while mitigating pathogen accumulation and autotoxicity [28]. This steering rests on three interdependent pillars of management (Figure 6).

### Pillar I: Engineering the plant partner (Holobiont Breeding)

Modern high‐yielding cultivars, historically bred for high‐input regimes, often lack the genetic architecture to foster beneficial microbiomes [248]. The new paradigm demands holobiont breeding, treating the crop and its microbiome as a single unit for genetic improvement. First, a clear understanding of the genetic mechanisms governing plant–microbiome interactions is required before selecting targets [83]. This goal leads to select for specific root architectures and exudate profiles that actively recruit beneficial taxa (Figure 6) [256]. For example, continuous cropping issues in cucumber are driven by *p*‐coumaric acid exudation, identifying a specific biochemical target for exclusion. Conversely, breeding can target the upregulation of strigolactone biosynthesis to enhance mutualist signaling (Figure 6) [97]. This pillar is operationalized by coupling root exudate metabolomics (LC‐MS/GC‐MS) with rhizosphere amplicon sequencing (16S rRNA/ITS2). GWAS and transcriptomics (RNA‐seq) can then functionally validate whether the selected cultivar is actively expressing traits that recruit target taxa like *Trichoderma* or AMF [79, 158]. For instance, transcriptomic profiling of root tissue under contrasting microbiome conditions provides functional validation via upregulation of defense‐suppressing or mutualist‐signaling pathways (*e.g*., strigolactone biosynthesis genes) [97]. These can also serve as a measurable indicator of successful holobiont trait expression. Empirical validation of this pillar requires demonstrating, across replicated field trials, that selected cultivars produce measurably altered exudate profiles and consistently recruit higher proportions of beneficial taxa as quantified by qPCR or metagenomic read mapping.

### Pillar II: Managing the soil habitat

Agronomic practices are the physical tools used to engineer a soil habitat that favors positive feedbacks. Mechanism behind this concept is as follows: Increasing spatial and temporal plant diversity (via complex rotations or intercropping) dilutes pathogen accumulation (Table 2 and Figure 6). Furthermore, strategic organic amendments can profoundly reshape the soil microbiome. For example, the innovative Livestock‐Crop‐Mushroom (LCM) circular system utilizes mushroom‐processed compost to actively suppress pathogen abundance by up to 36% while boosting oat biomass by 65% [335]. Reducing tillage can be empirically assessed by tracking hyphal length density (microscopy), glomalin‐related soil protein concentrations, and AMF colonization rates as quantitative proxies for preserved fungal network integrity [322]. Thus, conservation tillage further preserves the residual architecture of beneficial fungal networks (Figure 6) [322].

**Table 2:** A proposed model for adaptive plant‐soil feedback (PSF) management framework, pillars, and phases.

Management pillar	Molecular mechanism	Specific example
Partner engineering	Genetic and metabolic control of root exudates: Plants alter root exudate composition via specific signaling pathways in response to stress, which selectively recruits microbes.	In dense plantings, maize leaf volatile linalool activates the jasmonic acid pathway in roots, driving secretion of HDMBOA‐Glc. This compound reshapes the rhizosphere microbiome to enhance defense.
Managing the soil habitat	Pathogen subversion and plant counter‐defense: Pathogens use molecular tools to hijack plant signals, but plant genotype dictates the microbial recruitment outcome.	The fungal pathogen *Fusarium* produces fusaric acid, altering tomato root exudates. Resistant cultivars recruit beneficial bacteria in response, while susceptible ones do not.
Deploying biological agents	Metabolic interaction networks for syncom stability: The ecological stability of a Synthetic Community (SynCom) depends on the metabolic resource use patterns of its members.	Narrow‐spectrum resource‐utilizing bacteria (NSR) have limited carbon use but high metabolic interdependence. Incorporating NSR strains (e.g., *Cellulosimicrobium cellulans*) into SynCom enhances stability and plant growth promotion.

Phase	Core PSF concept	Agricultural translation and action
1. Diagnostic	Plants create species‐specific soil legacies.	**Map the feedback baseline:** Assess the soil's biological legacy from the previous crop (e.g., pathogen pressure, mutualist abundance) and soil health metrics (organic matter, nutrient status) before planning.
2. Intervention	PSF is driven by the balance of pathogens and mutualists.	**Apply the PSF toolbox:** Use agronomic practices to steer this balance. For example, break negative cycles with non‐host rotations or suppressive cover crops, and rebuild soil with diverse organic amendments to support mutualists.
3. Adaptive	Microbiomes dynamically modify their soil habitat, creating feedback loops.	**Close the management loop:** Monitor crop performance and soil biological shifts (e.g., via simple pathogen assays). Use this data to refine crop sequences and inputs, creating a farm‐specific learning cycle.

The efficacy of habitat management interventions can be measured through a multi‐omics soil monitoring framework. Shotgun metagenomics provides functional gene inventories (e.g., nitrogen cycling genes: *nifH*, *nirS*, *amoA*; phosphorus solubilization: *phoD*) that quantify changes in nutrient cycling capacity before and after rotation or amendment (Figure 6). Metabarcoding (ITS2 for fungi, 16S for bacteria) tracks community compositional shifts, with pathogen‐to‐mutualist ratios derived from taxonomic assignment serving as a key operational indicator of PSF trajectory. Soil metabolomics captures the net biochemical outcome, accumulation of allelopathic compounds (e.g., autotoxins in monocultures) versus beneficial exudates, providing a chemical fingerprint of feedback state (Figure 6). The LCM compost study exemplifies empirical validation at this scale [335]: treatment effects were validated by pairing microbial community profiling (ITS/16S amplicon sequencing [31]) with functional outcomes (pathogen suppression rates, biomass yield [250, 251]), establishing a cause‐effect chain that is both measurable and reproducible (Figure 6) [273].

### Pillar III: Deploying biological agents (Precision Inoculants)

When habitat management and plant genetics need a boost, direct biological interventions can be deployed. This goes beyond single‐strain inoculants towards consortia designed for function. The LCM system compost is itself a sophisticated multi‐species inoculant [335, 336, 337]. The future lies in developing microbial inoculants with high colonization efficiency and strong environmental adaptability tailored to specific crops and soil challenges. Furthermore, the concept of rewilding the plant microbiome (Figure 6) [248], by introducing microbial communities from wild or resilient plant relatives, is emerging as a strategy to restore lost protective functions (Figure 6) [33, 257]. The performance of biological agents can be evaluated through a tiered omics pipeline. First, inoculant establishment is confirmed using strain‐specific qPCR or amplicon sequence variant (ASV)‐level tracking in 16S/ITS datasets [338, 339], distinguishing introduced strains from indigenous taxa across time points. Second, functional activation is assessed by metatranscriptomics [340], which detects whether inoculated taxa are actively expressing target functions (e.g., antibiotic biosynthesis genes in *Pseudomonas* SynComs, such as *phlD* encoding 2,4‐diacetylphloroglucinol; siderophore genes *pvdA/pvdD*) rather than merely persisting (Figure 6). Third, network‐level integration is confirmed via co‐occurrence network analysis, which reveals whether introduced consortia displace or integrate within the existing microbial interaction network, a critical determinant of long‐term stability. Empirical validation requires longitudinal field trials that track inoculant persistence (qPCR‐quantified target gene copies g^−1^ dry soil), downstream community reorganization (beta‐diversity shifts), and critically agronomic endpoints (disease incidence, yield) to establish that biological activity translates into plant‐level outcomes. For SynCom deployment against soilborne pathogens, the ratio of suppressive taxa to target pathogen DNA (e.g., *Fusarium* spp., *Pythium* spp.) quantified by qPCR constitutes a practical, field‐deployable indicator of intervention success (Figure 6) [97, 98].

### A diagnostic cycle for PSF‐smart agriculture

Integrating these pillars requires a transition from generic agronomic recommendations to a dynamic, phased strategy: Diagnose, Intervene, and Adapt (Table 2 and Figure 6) [341].

1. Diagnosis (Reading the soil): Effective management begins with identifying the system's baseline state (Table 3). Is the soil suffering from a negative PSF (pathogen‐dominated/soil sickness), a neutral PSF (biologically degraded/low organic matter), or a Positive PSF (high mutualist activity)? Developing rapid, field‐deployable diagnostics, such as qPCR‐derived pathogen‐to‐mutualist ratios, is the immediate research frontier (Figure 6) [92].
2. Intervention (Layering the pillars): This diagnostic‐intervention framework represents a pivotal advancement for translating PSF theory into actionable farm management. It moves beyond generic recommendations to propose a dynamic, phased, and context‐adaptive strategy for steering soil biology toward resilience. The framework's power lies in its recognition that there is no single solution; effective management must first diagnose the system's state and then layer synergistic interventions across agronomic, genetic, and microbial levers (Figure 6).
  The core PSF principle is that soils can exist in different states driven by the historical balance of mutualistic and pathogenic organisms (Table 3). Negative PSF indicates a soil where host‐specific pathogens have accumulated, creating a soil sickness legacy that actively inhibits plant growth. Neutral/Low‐Function PSF describes a biologically degraded system with low organic matter and minimal microbial functional capacity, offering little feedback, positive or negative (Figure 6). Positive PSF is the target state, characterized by high mutualist activity, robust nutrient cycling, and disease suppression. The prescribed interventions are logically tailored to reverse, rebuild, or reinforce these distinct states (Table 3). Breaking a negative PSF requires habitat disruption (non‐host rotations, biofumigants) paired with host genetics that recruit antagonists (e.g., *Streptomyces*‐enriching tomatoes) [112, 232, 253]. Rebuilding a Neutral PSF relies heavily on Pillar II resource additions and Pillar I primed seeds to kickstart colonization. Thus, resource addition serves to create novel niches capable of restarting microbial life, while use of “primed” seeds or genotypes characterized by rich exudate profiles illustrates how plant‐driven signaling can feed nascent microbial communities, accelerating the transition from a pathogen‐dominated state to a balanced, suppressive soil microbiome (Table 3 and Figure 6) [33, 257]. Once a positive PSF is achieved, the goal shifts to maintenance and stability, and habitat management avoids disruptive actions, instead focusing on diversifying rotations and maintaining living cover to perpetuate the beneficial community [194, 198]. The final phase closes the loop, transforming this framework from a static prescription into a continuous learning system (Figure 6). This phase involves tracking key indicators (pathogen: mutualist ratios via qPCR, soil organic carbon, crop vigor) to answer two questions: Did the intervention shift the soil state as intended? Is the new state stable? The data feeds back to refine future diagnoses and intervention layers. For instance, if a pathogen‐suppressive SynCom fails to establish, the next cycle might prioritize a different habitat amendment or a more receptive plant cultivar. This adaptive cycle embodies the true application of PSF as a management tool, ensuring strategies evolve with the soil system they are designed to steward (Table 3 and Figure 6).
3. Adaptation (Digital integration): The final phase transforms the framework into a continuous learning system. By tracking key indicators (P:M ratios, soil carbon, crop vigor) [337], and integrating this data into farm management software, predictive algorithms can recommend optimized crop sequences based on a field's unique history (Figure 6) [24, 291].

**Table 3:** Additional drivers, tools, and layers of targeted Intervention Phase based on the diagnosis.

Feedback status	Primary tool (Habitat)	Secondary tool (Plant partner)	Tertiary tool (Microbial input)
**Negative PSF** (High pathogen load, low mutualists)	Break the cycle: Rotate with non‐host or suppressive cover crops (e.g., mustard for biofumigation). Reduce tillage to boost microbial diversity.	Deploy resistant cultivars bred to recruit antagonistic microbes (e.g., tomato with high *RIN* expression for *Streptomyces*) [53].	Apply targeted biocontrol consortia (e.g., *Pseudomonas* SynCom) to suppress pathogens.
**Neutral/Low‐Function PSF** (Low organic matter, degraded biology)	Rebuild habitat: Add diverse organic amendments (compost + crop residues). Integrate perennial or deep‐rooted cover crops.	Use microbiome‐priming seed coatings with beneficial endophytes. Plant genotypes with high exudate diversity.	Inoculate with mutualists (AMF, rhizobia) adapted to local soil conditions.
**Positive PSF** (High mutualist activity, good structure)	Maintain and diversify: Use complex crop rotations, maintain living cover. Avoid broad‐spectrum biocides.	Select for feedback stability; breed for traits that sustain mutualisms under stress.	Minimal intervention; monitor for shifts toward pathogen dominance.

The final, crucial step is the synergistic integration of these pillars (Table 2). An optimized crop rotation is the classic example of this integration in action [194, 198]: it uses habitat management (temporal diversification) for a selected plant partner (the crop variety) to leverage biological legacies (from the previous crop) for the benefit of the next (Figure 6). The ultimate aim is to design systems in which each component reinforces the others, creating a virtuous cycle that builds cultivated resilience, with farm productivity intrinsically linked to and supported by a healthy, self‐regulating soil ecosystem (Figure 6). This framework transforms PSF from an interesting observation into the central operating principle for a resilient agricultural system. It argues that the path to sustainable intensification is not through increasingly potent external inputs, but through the intelligent management of the biological interactions we now know how to predict and influence (Figure 6).

### Framework limitations and research frontiers

No conceptual framework is without boundaries. The three‐pillar model presented here is no exception, and three categories of limitation merit explicit acknowledgment.

Inter‐Pillar Antagonisms: The three pillars are not perfectly synergistic. For instance, conservation tillage (Pillar II) preserves AMF networks, but establishing a novel SynCom (Pillar III) may require strategic disturbance to lower colonization resistance. Similarly, breeding for mycorrhizal‐dependent phosphorus scavenging (Pillar I) inherently conflicts with high‐dose organic amendments that mineralize (Pillar II). These ecological trade‐offs must be explicitly managed within the adaptive cycle.

Stochastic Failure Modes: Predictive PSF models can fail when exogenous stochastic events override the conditioned community. Extreme climate shifts, novel invasive pathogens, or inadvertent pesticide misapplications that eliminate keystone taxa require models to include explicit uncertainty quantification and prediction intervals.

Contingency of the P:M Ratio: P:M ratio is a methodologically contingent metric, sensitive to sequencing depth, primer choice, and even diel sampling oscillations. Proposed diagnostic thresholds (e.g., >1.0 for negative feedback, <0.5 for positive feedback) must be treated as order‐of‐magnitude guides rather than absolute biological constants. Standardizing these protocols against massive, multi‐site yield data remains a paramount priority.

Ultimately, this framework argues that the path to sustainable agricultural intensification is not through increasingly potent external chemical inputs, but through the intelligent, data‐driven management of the biological interactions we now know how to predict and steer. Its value depends on sequencing depth, primer choice, rarefaction threshold, and sampling time (diel oscillations alone can shift relative abundances by measurable amounts). The diagnostic thresholds (>1.0 for negative feedback, <0.5 for positive feedback) should therefore be treated as order‐of‐magnitude guides rather than absolute cutoffs. Validation of these thresholds against crop yield and disease incidence data under standardized protocols remains a priority for future research. Collectively, these limitations do not invalidate the framework but instead define the conditions under which it can be applied reliably and highlight the research frontiers that must be addressed to move from descriptive patterns to predictive management.

## 10 FUTURE CHALLENGES

The synthesis of PSF principles represents a profound paradigm shift: moving away from reactive crop management toward the deliberate, predictive engineering of agroecosystems. However, the spatiotemporal organization of microbial function at the root‐soil interface is unresolved at the resolution required to identify causal functional zones. No integrated, empirically validated decision‐support framework currently exists that translates PSF diagnostics into dynamic management recommendations at the farm scale. Addressing these gaps requires the field to move beyond broad conceptual generalizations and target specific, mechanistic priorities grounded in testable hypotheses. While mGWAS can identify host loci that recruit beneficial taxa, we do not yet know the environmental plasticity of these traits. Can a cultivar bred to recruit *Streptomyces* in a temperate loam maintain that recruitment in a tropical clay, or is the extended phenotype strictly conditionally dependent? Future research must parameterize how abiotic stressors (drought, heat) silence or amplify plant‐driven microbial recruitment. We proposed rewilding the functional core and engineering vertical transmission to give crops an ecological priority effect. However, the long‐term stability of these engineered legacies remains unproven in open field conditions. Do seed‐borne endophytes persist until harvest, or are they eventually competitively excluded by the bulk soil microbiome?

We established that PSF stability is an emergent property of modular network topology and keystone taxa. Yet, current inoculant strategies still largely rely on flooding the system with massive doses of single strains. How do we design interventions that actively repair network topology? Can we identify the precise ecological disturbance required to open a niche for a keystone taxon without collapsing the existing beneficial network*?* Addressing these paradoxes requires the field to move beyond broad conceptual generalizations and target specific, mechanistic priorities grounded in testable hypotheses. A central challenge is moving past pairwise microbial effects to understand the higher‐order interactions among functionally complementary guilds. Priority must be given to dissecting the emergent dynamics between root‐colonizing PGPB, nutrient‐transforming microbes, and saprotrophic decomposers. Testable hypothesis 1: Engineered three‐guild SynComs, combining PGPB, nitrogen‐cycling bacteria, and saprotrophic fungi, will produce significantly stronger and more stable pathogen suppression than any pairwise combination. This stability, driven by emergent cross‐feeding and decomposer‐mediated pH modulation, will be quantifiable as lower pathogen DNA abundance (e.g., *Fusarium* copies g^−1^ soil) across diverse soil types.

Furthermore, future PSF frameworks must integrate the frequently overlooked top‐down regulators of the rhizosphere: soil viruses (bacteriophages) and protists. Bacteriophages are the most numerically abundant biological entities in soil, with estimated densities of 10^9^ virus‐like particles per gram, and their kill‐the‐winner dynamics, preferential lysis of numerically dominant bacterial strains, maintain bacterial diversity by preventing competitive exclusion [342, 343]. Whether specific phage communities in suppressive soils selectively lyse pathogenic bacteria while sparing slower‐growing mutualists (*Pseudomonas*, *Streptomyces*) remains a critical, agronomically consequential question. Protist Grazing: Phagotrophic protists (flagellates, amoebae, and ciliates) selectively graze bacteria, effectively mineralizing 20%–40% of rhizosphere bacterial nitrogen turnover [344, 345]. Management practices that alter soil aggregate structure directly modify this protist‐bacteria‐plant trophic cascade, implying that many PSF outcomes are driven by these microscopic predators.

A cross‐cutting methodological priority is the systematic transition from amplicon sequencing (which provides taxonomy but not functional reality) to integrated meta‐omics. Understanding the PSF state requires simultaneously capturing community potential (metagenomics), active expression (metatranscriptomics), translational output (metaproteomics), and the net chemical environment (metabolomics) [345, 346, 347]. Testable hypothesis 2: Metatranscriptomic profiling of the rhizosphere during the critical window of early root colonization (0–14 days post‐emergence) will identify a conserved suite of expressed functional genes that predicts final PSF direction with significantly greater accuracy than compositional metrics alone. Equally critical is mapping the micron‐scale spatial organization of these functions. Microbial location dictates activity. Testable hypothesis 3: Spatial metabolomics of the rhizosphere will reveal discrete, segregated zones of pathogen‐suppressive activity (e.g., phenazine and 2,4‐DAPG accumulation hotspots). The localization of these hotspots relative to the root tip will directly dictate the efficacy of targeted inoculant delivery systems (e.g., seed coating vs. furrow application).

Overcoming the strong context‐dependency of PSF requires the development of mechanistic machine‐learning models parameterized by four core tiers: plant traits (exudomics), dynamic abiotic constraints, microbial functional gene expression, and historical management legacies. Testable hypothesis 4: A multi‐tier predictive model integrating plant exudate metabolomics, functional metagenomics, and dynamic physicochemical variables will accurately forecast PSF direction (positive vs. negative) with ≥70% cross‐site classification accuracy, vastly outperforming taxonomy‐only models. These insights can directly inform practical interventions, including stage‐specific seed coatings, targeted inoculation strategies, and soil management practices designed to preserve critical microbial niches.

The ultimate translational output of these interconnected research priorities is the development of an integrated, predictive Decision‐Support Framework (DSF) specifically designed for PSF management. This tool must exist within the broader ecosystem of agricultural Decision Support Tools (DSTs), which are characterized by their diversity in models, systems, and digital implementations [337, 346, 347]. Existing DSTs typically aid decisions on specific aspects like nutrient balancing, organic matter turnover, or irrigation, often focusing on distinct soil functions [348].

Taken together, advancing PSF‐based agriculture is fundamentally an engineering challenge grounded in deep ecological mechanisms. By prioritizing higher‐order microbial consortia, spatially explicit functional mapping, and parameter‐rich predictive models, the next generation of agricultural microbiome research can deliver the actionable design principles required to cultivate truly resilient and sustainable agroecosystems.

## AUTHOR CONTRIBUTIONS

**Muhammad Asif**: Conceptualization (lead); writing—original draft (lead); writing— review and editing (lead); formal analysis (lead); visualizations (lead); investigations (lead). **Hazrat Bilal**: Writing— review and editing (supporting). **Min Xiao**: Writing— review and editing (supporting). **Lei Dong**: Writing— review and editing (supporting); validation(lead). **Wen‐Jun Li**: Supervision (lead), funding acquisition (lead).

## CONFLICT OF INTEREST STATEMENT

The authors declare no conflicts of interest.

## AI STATEMENT

The initial draft of this manuscript was polished for grammar and sentence structure using Gemini (3.1, Google Inc.). All AI‐generated contents were meticulously reviewed and edited by the authors to ensure factual accuracy and prevent any potential AI‐induced bias. We hereby confirm that no AI tools were employed in the processes of data generation, analyses, or visual content creation.

## ETHICS STATEMENT

No animals or humans were involved in this study.

## Data Availability

Data sharing is not applicable to this article as no datasets were generated or analyzed during the current study. No new data were generated or analyzed in this review. All information discussed in this article is derived from previously published studies cited in the reference list. Supplementary materials (graphical abstract, slides, videos, Chinese translated version, and updated materials) may be found in the online DOI or iMetaOmics Science http://www.imeta.science/imetaomics/.
